# Switching acidity on manganese oxide catalyst with acetylacetones for selectivity-tunable amines oxidation

**DOI:** 10.1038/s41467-019-10315-9

**Published:** 2019-05-28

**Authors:** Xiuquan Jia, Jiping Ma, Fei Xia, Mingxia Gao, Jin Gao, Jie Xu

**Affiliations:** 1 State Key Laboratory of Catalysis, Dalian National Laboratory for Clean Energy, Dalian Institute of Chemical Physics, Chinese Academy of Sciences, Dalian, 116023 China; 2 University of Chinese Academy of Sciences, Beijing, 100049 China

**Keywords:** Catalyst synthesis, Heterogeneous catalysis, Sustainability

## Abstract

The design of metal oxide catalysts predominantly focuses on the composition or geometry engineering to enable optimized reactivity on the surface. Despite the numerous reports investigating the surface chemisorption of organic molecules on metal oxides, insights into how adsorption of organic modifiers can be exploited to optimize the catalytic properties of metal oxides are lacking. Herein, we describe the use of enolic acetylacetones to modify the surface Lewis acid properties of manganese oxide catalysts. The acetylacetone modification is stable under the reaction conditions and strongly influences the redox-acid cooperative catalysis of manganese oxides. This enables a rational control of the oxidation selectivity of structurally diverse arylmethyl amines to become switchable from nitriles to imines.

## Abstract (web summary)

Exploiting the organic adsorbate-metal oxide interaction to optimize the catalytic properties of metal oxides has been rarely explored. Here, the authors use enolic acetylacetones to modify the surface Lewis acid properties of manganese oxide, enabling a rational control of the oxidation selectivity of structurally diverse arylmethyl amines.

## Introduction

Enhanced control of selectivity has been a key focus in catalysis science^1–7^. In the past few years, the excitement that surrounds the discovery of selectivity-tunable chemical processes was stoked by the heterogeneous catalysts with precisely controllable reactivities^8–14^. Still, repetitive and exhausting engineering of the composition or geometry of catalysts is frequently required to switch selectivity in the case of the consecutive reaction generating more than one compound of importance. Apart from alteration of the intrinsic characteristics of catalysts, surface modification provides a facile way to switching selectivity to different products over the same catalyst by controlling the reactant orientation or availability of specific sites on the catalyst surface^4,8,15,16^. Owing to the fascinating flexibility of organic modifiers, organic modification has evolved into very powerful and effective technique for the selectivity control of supported metal catalysts^2,4,8,17–21^. Much less is known about the study on tuning the selectivity of metal oxide catalyst via organic modification^22,23^, although metal oxides are used in various fields of bifunctional catalysis as a result of their acid–base and redox properties with high thermal stability and durability^24–28^.

Organic adsorbates on metal oxides were extensively probed for insights into the mechanistic chemistry. By contrast, how the organic adsorbate-metal-oxide interaction can be exploited to optimize the catalytic properties of metal oxides remains unclear, which would be more attractive from the viewpoint of practical chemistry. In this study, the selective oxidation of primary amines was chosen as the model consecutive reaction for studies on the effect of organic modification on the selectivity switch of metal oxide catalysts. The dual functions of both Lewis acid and redox properties make manganese oxides (MnO_x_) a promising catalyst for the aerobic oxidation of amines to obtain imines, nitriles or amides via oxidative dehydrogenation and successive hydrolysis^29–31^. On the other hand, the selective synthesis of imines or nitriles via this bifunctional catalysis process is still challenging. A general feature of such reactions is the reversible and uncontrollable hydrolysis of the C=N bond in aldimine intermediates or imines promoted by the Lewis acidity of MnO_x_^29,30,32^. Developing ways to control the cooperations between the redox and acid properties on MnO_x_ catalysts is therefore worthwhile.

We have now made such catalysts by employing amorphous MnO_x_ as redox-acid cooperative catalyst for the double dehydrogenation of primary amines to nitriles, and the acetylacetone (acac) modifier switches the selectivity from nitriles to imines by selectively suppressing the Lewis acidic sites on MnO_x_ catalyst. This work provides an opportunity for using manganese oxide catalyst for the synthesis of both imines and nitriles in high selectivity from primary amines via aerobic oxidation, and contributes an example of regulating the catalytic selectivity of metal oxide catalysts by organic modification on the surface.

## Results

### Aerobic oxidation of primary amines over manganese oxides

**Fig. 1:**
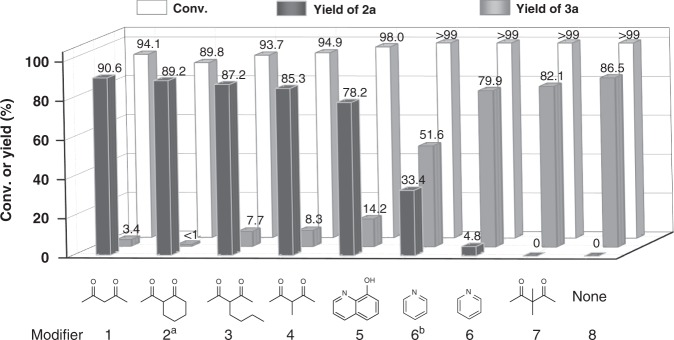
Performance of MnO_x_ with different modifiers in the aerobic oxidation of benzylamine. Reaction conditions: 1 mmol benzylamine, 0.1 mmol MnO_x_, additive/MnO_x_ = 20 mol%, 5 mL CH_3_CN, 0.3 MPa O_2_, 90 °C, 14 h. ^a^20 h. ^b^Additive/MnO_x_ = 200 mol%

The aerobic oxidation of benzylamine was selected as a model reaction. As shown in Table 1, using well-crystallized MnO_2_ catalysts, a near quantitative conversions were observed, and the yields of nitrile (**3a**) varied from 24.6% to 58.9%, with 24.1–61.9% yield of imine (**2a**) formed (Table 1, entries 1–4). In comparison, amorphous MnO_x_ has much increased specific surface area and mass-specific activity (Supplementary Table 1)^23^, affording a complete conversion of amine and 86.5% yield of nitrile (**3a**) (Table 1, entry 5). The time course of the synthesis of **3a** over amorphous MnO_x_ displays a steep volcano curves for **2a**, indicating that the imine of **2a** is the reaction intermediate and could be hardly obtained in high selectivity via tuning of the reaction time (Supplementary Fig. 1). Benzamide was detected as the main byproduct (Supplementary Fig. 7). Accordingly, the double dehydrogenation reaction of the primary amines to nitriles was more favorable in the case of using amorphous MnO_x_ catalyst in comparison with the studied crystallized MnO_2_. Next, we explored the possibility of enhancing the selectivity for **2a** over MnO_x_ catalyst. By reducing the reaction temperature or ratio of the catalyst to the substrate, ~46.7% yield of **2a** was obtained as the highest yield (Fig. 1, entries 6–7). Notably, when the MnO_x_ catalyst was modified with acac by immersing the catalyst in an acetonitrile solution of acac and stirring at 90 °C for 4 h, the second dehydrogenation step of imine oxidation was prohibited, and a dramatic change in selectivity from nitrile toward imine product was observed (Table 1, entry 8; Supplementary Figs. 1 and 8). However, the mass-specific activity was decreased after modification. Thus, the switched selectivity was not because of an increase in the rate of the desired reaction but rather a result of inhibition of the undesired reaction for the modified catalyst.

**Table 1:** The catalytic performance of manganese oxide catalysts in the benzylamine aerobic oxidation reaction


Entry	Cat.	Mass-specific activity (mmol g_cat_^−1 ^ h^−1^)	Conv. (%)	Yield (%)	
				2a	3a
1	α-MnO_2_	45.4 ± 2.1	94.5 ± 0.4	47.9 ± 2.4	44.6 ± 3.1
2	γ-MnO_2_	61.8 ± 4.0	97.4 ± 0.2	58.6 ± 4.4	32.5 ± 3.7
3	δ-MnO_2_	15.7 ± 0.7	94.9 ± 0.1	61.9 ± 3.0	24.6 ± 5.0
4	OMS-2	63.4 ± 5.2	93.1 ± 0.2	24.1 ± 2.6	58.9 ± 2.0
5	MnO_x_	129.2 ± 4.2	>99	0	86.5 ± 0.3
6^a^	MnO_x_	—	>99	7.0 ± 1.2	80.1 ± 1.0
7^a, b^	MnO_x_	—	93.0 ± 0.2	46.7 ± 0.2	41.2 ± 2.5
8	acac-MnO_x_	14.7 ± 1.9	94.1 ± 0.3	90.6 ± 4.1	3.4 ± 0.2

Reaction conditions: 1 mmol Benzylamine, 0.1 mmol catalyst, acac/MnO_x_ = 20 mol% for acac-MnO_x_, 5 mL CH_3_CN, 0.3 MPa O_2_, 90 °C, 14 h.

^a^0.05 mmol MnO_x_

^b^60 °C

Acac and its derivatives are particularly attractive in the functionalization of metal oxide nanoparticles for its capability to form stable adsorption on metal oxide surfaces^33–35^. As shown in Fig. 1, the MnO_x_ modified by 2-acetylcyclohexanone (**2**), 3-butyl-2,4-pentanedione (**3**) or 3-methyl-2,4-pentanedione (**4**) produced imine with a similar yield of ~90%. These results suggest that the acac derivatives with different substituent groups are effective modifiers for tuning the selectivity. Modification with pyridine (**6**) just provided a slight increase in the yield of imine relative to that of clean MnO_x_. And moderate yield of imine was observed upon increasing the amount of pyridine by 10 times. By contrast, 8-hydroxyquinoline (**5**) gave an obvious increase in the yield of imine, possibly due to the higher stability of bidentate coordination in comparison with the monodentate pyridine modifiers. Moreover, surface coverages of 5–6 μmol m^−2^ were observed for the modifiers of acac (**1**), 2-acetylcyclohexanone (**2**), 3-butyl-2,4-pentanedione (**3**), 3-methyl-2,4-pentanedione (**4**), and 8-hydroxyquinoline (**5**) (Supplementary Figs. 2 and 3). By contrast, a considerably low surface coverage was observed over pyridine (**6**) (Supplementary Fig. 2). This is in accordance with the performance of MnO_x_ modified by these modifiers in the aerobic oxidation of benzylamine. For β-dicarbonyl compounds, a keto-enol tautomerism usually exists^36–38^. And the reaction pathways over the MnO_x_ catalyst might be directed by the keto form or enolic form of acac. 3,3-Dimethyl-2,4-pentanedione (**7**), lacking the proton required to generate the enol form of β-diketones^39^, minimally affected the selectivity relative to that of clean MnO_x_, which differs especially from the other acac derivatives. In addition, the surface coverage of 3,3-dimethyl-2,4-pentanedione (**7**) is the lowest among the tested modifiers (Supplementary Figs. 2 and 4). Thus, we deduce that, during the self-assembly process, the enolic form of acac rather than the keto is the indispensable species for regulating the surface properties of MnO_x_ which gets the catalytic selectivity to switch from nitrile to imine.

### Effect of acac modification on surface properties of MnO_x_

**Fig. 2:**
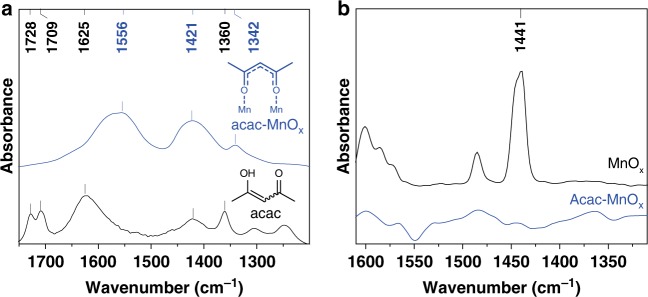
Catalyst characterization. **a** FT-IR spectra of acac and acac-MnO_x_ after evacuation at 150 °C for 0.5 h. **b** FT-IR spectra of pyridine adsorption to MnO_x_ and acac-MnO_x_

Next, the mode of acac adsorption as well as the resulting surface regulation effect on MnO_x_ was studied. The self-assembly of acac on the surfaces of MnO_x_ was characterized by Fourier transform infrared (FT-IR) spectroscopy (Fig. 2a). In order to probe whether coordination interaction occurred between acac and the MnO_x_ surface, we have compared the IR spectroscopy of free acac and acac modified MnO_x_. For free acac, two C=O stretching bands at 1728 and 1709 cm^−1^, one C=C stretching band at 1625 cm^−1^ and two CH_3_ bending vibration bands at 1421 and 1360 cm^−1^ were observed, all of which are attributed to enolic acac with varied conformations^40^. After the acac deposition on MnO_x_, the C=O stretching bands attributed to free acac disappeared, and two bands were observed at 1556 and 1342 cm^−1^ that arise from the metal-complexed CO/CC stretching and CH_3_ symmetrical bending vibration of coordinated acac, respectively^41,42^. This indicates the formation of bidentate ligand from the enol form of acac via losing its proton and subsequent coordination on the surface of MnO_x_. The retainment of the vibration peaks after evacuation at 150 °C for 0.5 h established the stability of acac modification.

To examine the effect of acac modification on the surface properties of MnO_x_, in situ FT-IR spectroscopic characterization of pyridine adsorption was performed (Fig. 2b). The significant band at 1440 cm^−1^ was observed in the spectrum of unmodified MnO_x_, assigned to coordinatively bound pyridine on Lewis acidic sites, indicating that the unmodified MnO_x_ surface is covered by Lewis acidic sites^27,43^. By contrast, after pre-adsorption of acac on MnO_x_, the band at 1440 cm^−1^ was scarcely observed under identical conditions. These results demonstrate that the Lewis acidic sites on the surface of MnO_x_ can be blocked by pre-adsorption of acac modifier.

It is reasonable to expect the retention of Lewis acidity in those modified catalysts which showed low selectivity for imine. As shown in Supplementary Fig. 5, in situ FT-IR spectroscopic characterization of pyridine adsorption confirmed the preservation of Lewis acidity on 3,3-dimethyl-2,4-pentanedione (**7**) modified MnO_x_, which gave nitrile as the primary product in the aerobic oxidation of benzylamine (Fig. 1). To examine the acid property of catalysts showing intermediate selectivity, MnO_x_ modified with a decreased amount of 10 mol% acac was prepared and tested. A residual Lewis acidity was observed for 10 mol% acac modified MnO_x_ (Supplementary Fig. 5), which afforded a medium yield (51.0%) of imine (**2a**) under identical conditions (Supplementary Fig. 6).

### Understanding the switchable selectivity over MnO_x_ catalyst

**Fig. 3:**
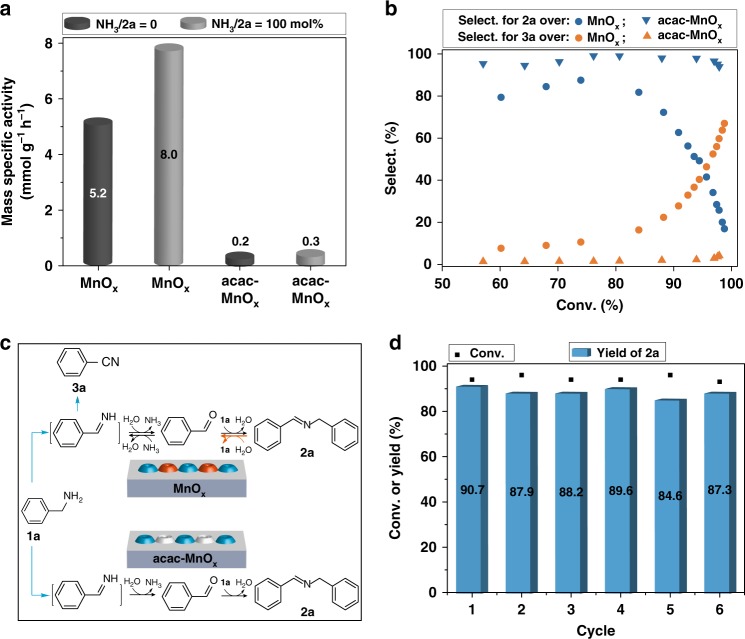
Reactivity of *N*-benzylidenebenzylamine (**2a**) over unmodified and acac-modified MnO_x_. **a** Mass-specific activities (mmol g_cat_^−1^ h^−1^) of MnO_x_ and acac-MnO_x_ in aerobic oxidation/ammoxidation of N-benzylidenebenzylamine (**2a**) under 0.3 MPa O_2_ at 90 °C. The mass-specific activity was measured at the reaction time of 1 h when the conversion is below 30%. **b** Selectivity-conversion correlation of N-benzylidenebenzylamine (**2a**) in aerobic oxidation of benzylamine under 0.3 MPa O_2_ at 90 °C. **c** Proposed mechanism. **d** Recyclability test results for selective synthesis of *N*-benzylidenebenzylamine (**2a**) by oxidation of benzylamine over acac modified MnO_x_. Reaction conditions: 1 mmol benzylamine, 0.1 mmol MnO_x_, acac/MnO_x_ = 20 mol%, 5 mL CH_3_CN, 0.3 MPa O_2_, 90 °C, 14 h

If the availability of Lewis acid sites on a metal oxide surface can be controlled, the reaction promoted by the acidity of the catalyst can be intentionally tuned. It is known that the oxidation reaction of imines was initiated by their fragmentating into aldehydes and amines via hydrolysis^32,44^. And the activity for hydrolysis reaction over manganese oxide catalysts is closely related to the Lewis acidity on the catalyst surface^30,45^. Most likely, the change of Lewis acid sites availability via acac modification on the surface of MnO_x_ catalysts should directly influence the reactivity of imines. To test this hypothesis, we subsequently examined the effect of acac surface modification on the oxidative reactivity of imine (Fig. 3a). Unmodified MnO_x_ showed obvious activity based on the **2a** conversion (5 mmol g_cat_^−1^ h^−1^ for aerobic oxidation, 8 mmol g_cat_^−1^ h^−1^ for aerobic ammoxidation). Notably, the acac-modified catalysts exhibited a distinct decrease in activities for both **2a** oxidation and ammoxidation. Consequently, the MnO_x_ with inhibited acid properties via acac modification contributed to the extinguished reactivity of imine.

To obtain a better understanding of the role of acac modification in the primary amine oxidation reaction catalyzed by MnO_x_, we analyzed the catalytic selectivity in the aerobic oxidation of benzylamine over a broad range of conversions (Fig. 3b). It is widely accepted that the oxidation of benzylamine initially generates aldimine as the intermediate product, which can be hydrolyzed or directly oxidized according to the redox or coordinative properties of catalysts^11^. Herein, unmodified MnO_x_ gave **2a** in the selectivity of around 80% at the conversion below 80%. Concomitantly, a steadily increased selectivity for **3a** was observed with increasing the conversion. This indicated that hydrolysis of aldimine occurred as the main reaction to give aldehyde instead of the direct oxidation to generate **3a**. And the imine of **2a** should be formed via the facile condensation reaction of benzaldehyde with benzylamine. Meanwhile, in situ formed **2a** was hydrolyzed back to form aldimine, followed by oxidation to **3a**. Remarkably, at the conversion higher than 80%, the selectivity for **2a** rapidly decreased, accompanied by a significant increase in the selectivity for **3a**, rationalizing the reaction pathway shown in Fig. 3c. This indicated that the reversibility of the condensation reaction step between benzaldehyde and benzylamine directly influenced the resultant selectivity. By contrast, **3a** was scarcely formed over acac-modified MnO_x_ at the conversions ranging from 50 to 98%, and the selectivity for **2a** is >90%, indicating a highly favorable active-site selection effect of acac modifiers. Thus, after inhibition of the reactivity of imine, acac modified MnO_x_ catalyst exhibited only specific types of sites required for aerobic dehydrogenation-coupling of benzylamine (**1a**) to form imine.

One of the central issues challenging the continued development and refinement of this surface modification technique concerns the stability of the organic modification under demanding reaction conditions. In order to examine the catalytic performance over extended periods of time, we attempted to recycle the catalyst without regeneration. As shown in Fig. 3d, the catalyst can be recycled for at least 5 times to give a TON of 57 without selectivity loss, suggesting that the regulated surface properties of acac-modified catalyst are not destroyed under the reaction conditions. This should be ascribed to the ability of acac to form stable coordination complexes on the surface of MnO_x_.

### Synthesis of nitriles and imines

Figure 4 shows data for aerobic oxidation of various substituted benzylamines (see Supplementary Table 2 and Supplementary Figs. 9–38 for detailed data) on MnO_x_ and acac-MnO_x_ catalysts, respectively. The MnO_x_ catalyst is generally active and selective for the nitriles, while the acac-MnO_x_ catalyst is highly selective for the imines. In the presence of MnO_x_ catalyst, benzylamine derivatives bearing electron-donating groups gave the corresponding nitriles in relative higher yields in comparison with those bearing electron-withdrawing groups, due to the facile hydrolysis reaction of electron-withdrawing groups substituted nitriles to amides. For acac-MnO_x_ catalyst, benzylamine derivatives bearing both electron-donating and electron-withdrawing groups reacted to produce the corresponding imines in good to excellent conversions and yields.

**Fig. 4:**
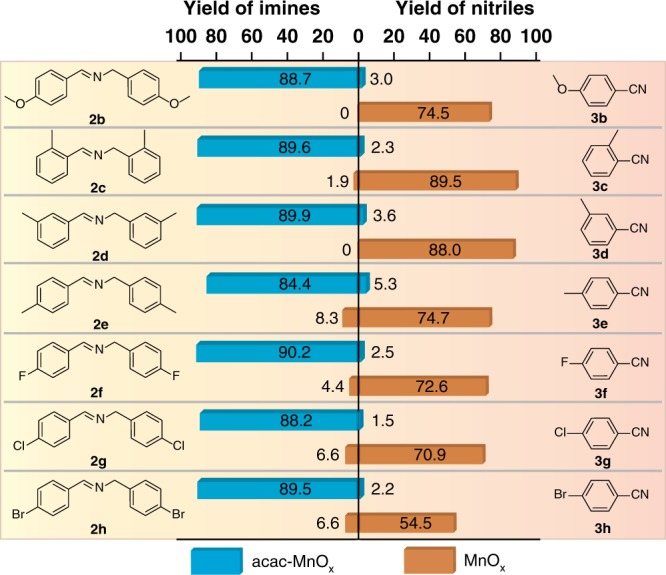
Synthesis of nitriles and imines using benzylamine derivatives over unmodified and acac-modified MnO_x_. Reaction conditions: 1 mmol benzylamine, 0.1 mmol MnO_x_, acac/MnO_x_ = 20 mol% for acac-MnO_x_, 5 mL CH_3_CN, 0.3 MPa O_2_, 90 °C, 14 h

## Discussion

In summary, we have demonstrated a method of using organic modifiers to tune the reaction pathway of redox-acid catalysis on the surface of metal oxide catalysts. Upon modification with enolic acetylacetones, the selectivity for manganese oxide catalyzed primary amines oxidation reaction switched from nitriles to imines. The acetylacetone modification is stable under the reaction conditions and demonstrated good recyclability. The current study opens the door to the development of a class of highly stable and selectivity-switchable metal oxide catalysts via using the versatility of organic ligands to tune the surface properties of metal oxide catalysts.

## Methods

### Preparation of MnO_x_

MnO_x_ was prepared according to the literature procedure^23^. A 100 mL aqueous solution containing 40 mmol KMnO_4_ was added into another 250 mL solution of EtOH-H_2_O (5:1) containing 40 mmol MnAc_2_. After addition complete, adjust the pH to 8 with aq. NH_3_, then the mixture was stirred at room temperature for 12 h, the resulting solid was collected by filtration, washed repeatedly with distilled water, and finally dried for 48 h in air at 80 °C.

### Preparation of organic modified MnO_x_

Surface organic modified catalysts were prepared by immersing the catalyst in an acetonitrile solution of modifiers. After stirring at 90 °C for 4 h, the mixture of modifier solution and the catalyst was used for reaction without further separation.

### Catalyst characterization

In situ FT-IR spectra of the acac-MnO_x_ and pyridine adspecies on the catalysts were recorded with a TENSOR 27 spectrometer equipped with an in situ IR cell connected to a conventional gas flow system. The samples (20–30 mg) were pressed into self-supporting wafers (20 mm in diameter) and mounted in the IR cell. The adsorption of acetylacetone on MnO_x_ was characterized with the following method: The acac-MnO_x_ sample was pretreated at 150 °C under vacuum (<10^−1^ Pa) for 30 min. After cooling to 30 °C, IR measurements were carried out. The adsorption of pyridine was carried out with the following method: The sample was pretreated at 150 °C under vacuum (<10^−1^ Pa) for 30 min. After cooling to 30 °C, pyridine was fed into the **in situ** IR cell under vacuum (<10^−1^ Pa). Then, the sample was heated at 150 °C for 30 min to remove the physical adsorbed pyridine. And IR measurements were carried out after cooling to 30 °C.

### General experimental procedures

Catalytic reactions were performed in a 20 mL stainless-steel autoclave equipped with a magnetic stirrer, a pressure gauge, and automatic temperature control apparatus. The reactor was connected to an oxygen cylinder for reaction pressure. In a typical experiment, benzylamine (107.5 mg, 1 mmol) and the prepared suspension of organic modified MnO_x_ were loaded into the reactor. After sealing and charging with O_2_ (0.3 MPa), the autoclave was heated to the desired temperature (90 °C). After reaction, the autoclave was cooled. The solution was separated by centrifugation and analyzed by GC using the internal standard method. The error bars (standard deviation) were calculated from repeat measurements.

The products were identified by Agilent 6890 N GC/5973MS as well as by comparison with the retention times to corresponding standards in GC traces. Gas chromatography measurements were conducted on Agilent 7890 A GC with autosampler and a flame ionization detector. DB-17 capillary column (30 m × 320 μm × 0.25 μm) was used for separation of reaction mixtures. The temperature of the column was kept at 100 °C for 3 min, then increased to 280 °C at a rate of 15 °C min^−1^ and kept for 6 min. The conversion of benzylamine and yield of corresponding products were evaluated using naphthalene as the internal standard. The conversion of other substrates and yield of corresponding products were determined based on area normalization without any purification.

## Supplementary information

Supplementary Information

Peer Review

## Data Availability

All data generated and analyzed during this study are included in this Article and its Supplementary Information or are available from the corresponding author upon reasonable request.
