# Supplementary material for: Switching acidity on manganese oxide catalyst with acetylacetones for selectivity-tunable amines oxidation
Source: Nat Commun. 2019 May 28;10:2338. doi: 10.1038/s41467-019-10315-9 (PMC6538668; doi:10.1038/s41467-019-10315-9)
Supplement: Supplementary file 1 — Supplementary Information [file 41467_2019_10315_MOESM1_ESM.pdf]

Supplementary Information

Switching Acidity on Manganese Oxide Catalyst with Acetylacetones for Selectivity-tunable Amines Oxidation

Jia et al.

Supplementary Information

Supplementary Methods 3
Supplementary Table 1 4
Supplementary Figure 1 4
Supplementary Figure 2 5
Supplementary Figure 3 5
Supplementary Figure 4 6
Supplementary Figure 5 6
Supplementary Figure 6 6
Supplementary Table 2 7
Supplementary Figure 7 8
Supplementary Figure 8 8
Supplementary Figure 9 9
Supplementary Figure 10 9
Supplementary Figure 11 10
Supplementary Figure 12 10
Supplementary Figure 13 11
Supplementary Figure 14 11
Supplementary Figure 15 12
Supplementary Figure 16 12
Supplementary Figure 17 13
Supplementary Figure 18 13
Supplementary Figure 19 14
Supplementary Figure 20 14
Supplementary Figure 21 15
Supplementary Figure 22 15
Supplementary Figure 23 16
Supplementary Figure 24 16
Supplementary Figure 25 17
Supplementary Figure 26 17
Supplementary Figure 27 18
Supplementary Figure 28 18
Supplementary Figure 29 19
Supplementary Figure 30 19
Supplementary Figure 31 20
Supplementary Figure 32 20
Supplementary Figure 33 21
Supplementary Figure 34 21
Supplementary Figure 35 22
Supplementary Figure 36 22
Supplementary Figure 37 23
Supplementary Figure 38 23

Supplementary Methods

Materials. All the chemicals used were of analytical grade and used as received unless otherwise stated. CH_3CN, pyridine, 8-hydroxyquinoline, $KMnO_4$ and $MnAc_2$ were purchased from Tianjin Kermel Chemical Reagent Co. Ltd. Benzonitrile and acetylacetone (acac) was purchased from Sinopharm Chemical Reagent Co., Ltd. 4-Methylbenzylamine, 3-methylbenzylamine, 4-methoxybenzylamine and 4-fluorobenzylamine were obtained from Aladdin Chemistry Co. Ltd. 2-Acetylcyclohexanone was purchased from TCI Shanghai. γ-MnO_2, 3-methyl-2,4-pentanedione and 3-butyl-2,4-pentanedione were purchased from Alfa Aesar. 2-Methylbenzylamine, 4-bromobenzylamine and 4-chlorobenzylamine were purchased from Energy Chemical. Benzylamine, N-benzylidenebenzylamine and 3,3-dimethyl-2,4-pentanedione were obtained from Sigma-Aldrich.

Preparation of α-MnO_2. α-MnO_2 was prepared according to the literature procedure.[1] A 100 mL aqueous solution containing 40 mmol $KMnO_4$ and 108 mmol NaOH was added into another 100 mL aqueous solution containing 60 mmol $Mn(NO_3)_2$. After stirring at room temperature for 4 h, the resulting solid was collected by filtration, washed repeatedly with distilled water, and finally dried overnight in air at 60 °C and then calcined in air at 500 °C for 4 h.

Preparation of δ-MnO_2. δ-MnO_2 was prepared according to the literature procedure.[2] An aqueous solution (A) was prepared by dissolving 0.04 mol of $KMnO_4$ and 1.2 mol of NaOH in 400 mL of water. Afterward, another aqueous solution (B) was prepared by dissolving 0.112 mol of $MnCl_2\cdot 4H_2O$ in 400 mL of water. Solution B was added dropwise to solution A with vigorous stirring in an ice bath. The resulting precipitate was statically aged at room temperature for 1 day and then washed and dried at 90 °C.

Preparation of OMS-2. OMS-2 was prepared according to the literature procedure.[3] $KMnO_4$ (5.89 g) in water (100 mL) was added to a solution of $MnSO_4\cdot H_2O$ (8.8 g) in water (30 mL) and conc. HNO_3 (3 mL). The solution was refluxed at 100 °C for 24 h. Then, the dark brown solid was filtered off, washed with a large amount of water (ca. 3 L), and dried at 120 °C to afford 8.2 g of OMS-2.

Supplementary Equations:

$$\text{Conversion} = \left(1 - \frac{\text{Moles of substrate}}{\text{Moles of substrate loaded initially}}\right) \times 100\% \quad (1)$$

$$\text{Selectivity of product} = \left(\frac{\text{Moles of product}}{\text{Moles of substrate converted}}\right) \times 100\% \quad (2)$$

$$\text{Mass specific activity} = \frac{\text{Moles of substrate converted}}{\text{Mass of catalyst} \times \text{Reaction time}} \quad (3)$$

$$\text{Surface specific activity} = \frac{\text{Mass specific activity}}{\text{Specific surface area of catalyst}} \quad (4)$$

Supplementary Table 1 The result of surface area and activity for MnO_x vs. crystalline MnO_2.

Entry	Cat.	BET surface area ($m^2\ g^{-1}$)	Mass-specific activity ($mmol\ g_{cat}^{-1}\ h^{-1}$)	Surface-specific activity ($mmol\ h^{-1}\ m^{-2}$)
1	α-MnO_2	28.2	45.4±2.1	1.6±0.1
2	γ-MnO_2	69.5	61.8±4.0	0.9±0.1
3	δ-MnO_2	49.7	15.7±0.7	0.3
4	OMS-2	77.4	63.4±5.2	0.8±0.1
5	MnO_x	250.4	129.2±4.2	0.5

The error bars (standard deviation) were calculated from repeat measurements.

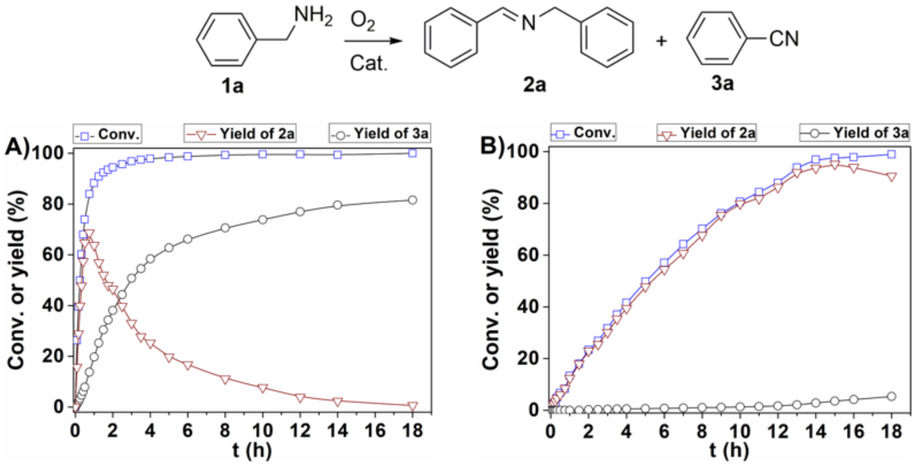

Supplementary Figure 1 Time course of the aerobic oxidation of benzylamine. A) MnO_x as catalyst. B) acac-modified MnO_x as catalyst. Reaction conditions: 1 mmol benzylamine, 0.1 mmol MnO_x, acac/MnO_x = 20 mol% for acac-modified MnO_x, 15 mL CH_3CN, 0.3 MPa O_2 at 90 ^oC.

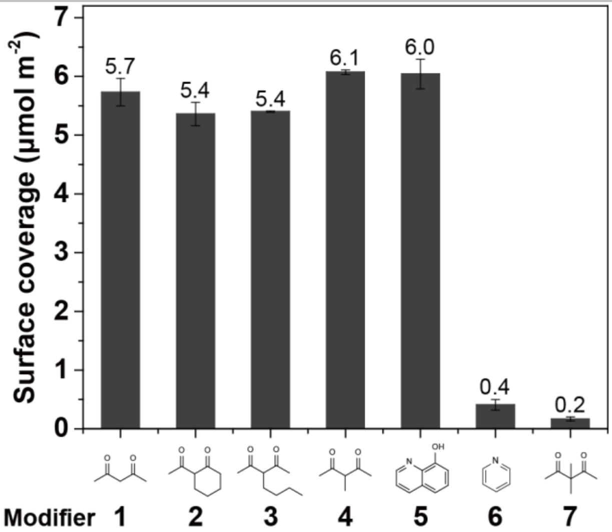

Supplementary Figure 2 Surface coverage of the organic modifiers**.** Process for evaluating the modifier adsorption on MnO_x: Immersing 0.1 mmol MnO_x in a 5 mL acetonitrile solution of modifiers (modifier/MnO_x = 20 mol%). After stirring at 90 ^oC for 4 h, the excess of unadsorbed modifier in solution was determined by GC analysis. The error bars (standard deviation) were calculated from repeat measurements.

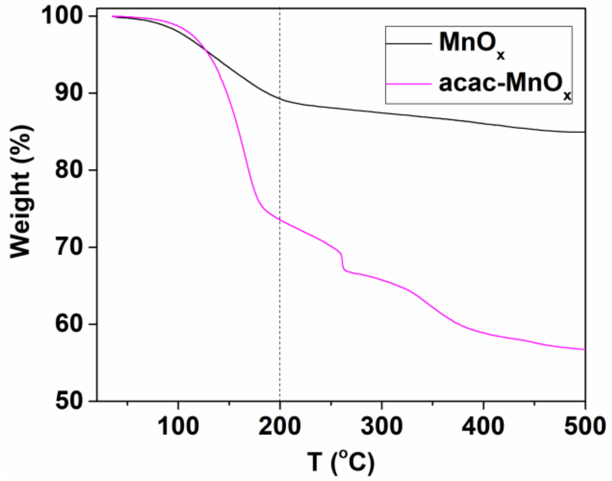

Supplementary Figure 3 TGA profiles of MnO_x and acac-MnO_x, respectively**.** According to the TGA profiles, the mass fraction of chemically adsorbed acac was obtained to be 13.1%. And the surface coverage of chemically adsorbed acac could be further calculated to be 6.0 μmol m^{-2}.

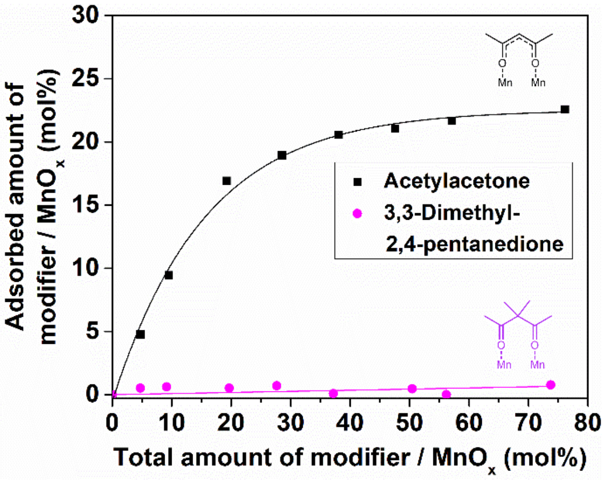

Supplementary Figure 4 The chart of the acetylacetone and 3,3-dimethyl-2,4-pentanedione adsorption on MnO_x. Process for evaluating the modifier adsorption on MnO_x: Immersing 0.1 mmol MnO_x in a 5 mL acetonitrile solution of modifiers. After stirring at 90 °C for 4 h, the excess of unadsorbed modifier in solution was determined by GC analysis.

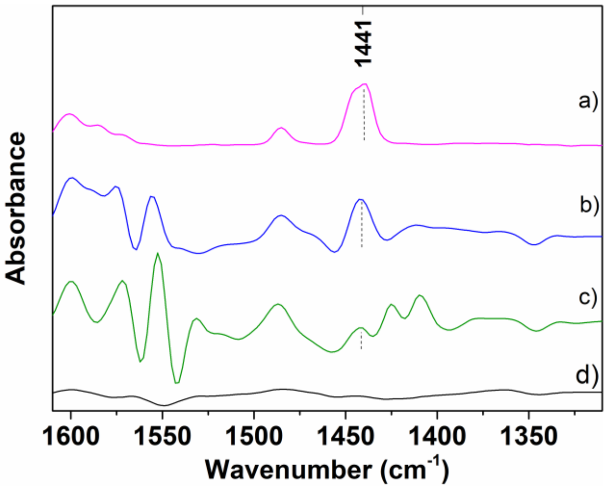

Supplementary Figure 5 FT-IR spectra of pyridine adsorption to a) clean MnO_x, b) 20 mol% 3,3-Dimethyl-2,4-pentanedione modified MnO_x, c) 10 mol% acac modified MnO_x and d) 20 mol% acac modified MnO_x.

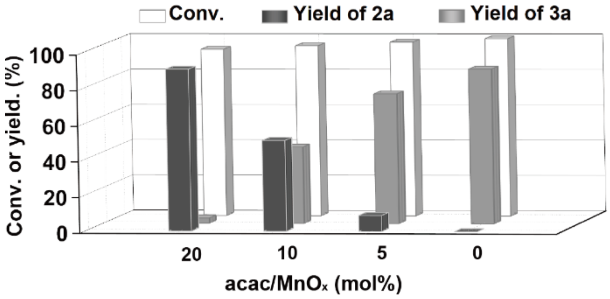

Supplementary Figure 6 Performance of MnO_x with different amount of acac modifier in the aerobic oxidation of benzylamine. Reaction conditions: 1 mmol benzylamine, 0.1 mmol MnO_x, 5 mL CH_3CN, 0.3 MPa O_2, 90 °C, 14 h.

Supplementary Table 2 The catalytic performance of manganese oxide catalysts in the substituted benzylamine aerobic oxidation reaction. [a]

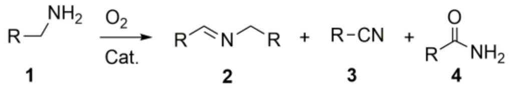

Entry	Substrate	Cat.	Conv. [%]	Yield [%]		
				**2**	**3**	**4**
1	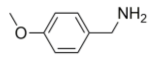	MnO_x	>99	0	75	11
2		acac-MnO_x	93	89	3	-
3	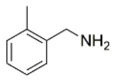	MnO_x	>99	2	89	4
4		acac-MnO_x	96	90	2	-
5	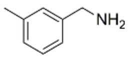	MnO_x	>99	0	88	11
6		acac-MnO_x	96	90	4	-
7	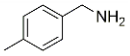	MnO_x	>99	8	75	9
8		acac-MnO_x	94	84	5	-
9	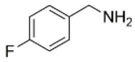	MnO_x	>99	4	73	18
10		acac-MnO_x	95	90	2	-
11	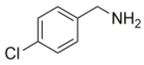	MnO_x	>99	7	71	20
12		acac-MnO_x	92	88	2	-
13	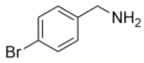	MnO_x	>99	7	55	32
14		acac-MnO_x	93	89	2	-

[a] Reaction conditions: 1 mmol amine, 0.1 mmol MnO_x, acac/MnO_x = 20 mol% for acac-MnO_x, 5 mL CH_3CN, 0.3 MPa O_2, 90 °C, 14 h.

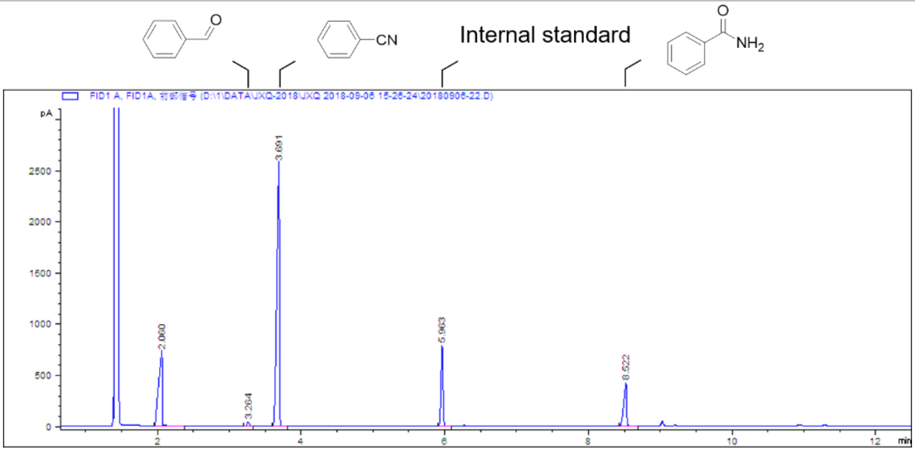

Supplementary Figure 7 GC spectrum of products from aerobic oxidation reaction of benzylamine over MnO_x (**Table 1, entry 5** in the main text). Reaction conditions: 1 mmol benzylamine, 0.1 mmol MnO_x, 5 mL CH_3CN, 0.3 MPa O_2, 90 °C, 14 h.

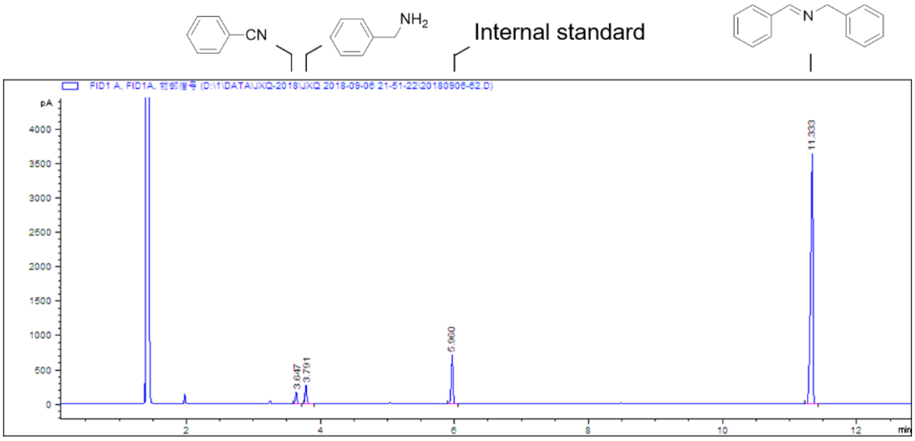

Supplementary Figure 8 GC spectrum of products from aerobic oxidation reaction of benzylamine over acac-MnO_x (**Table 1, entry 8** in the main text). Reaction conditions: 1 mmol benzylamine, 0.1 mmol MnO_x, acac/MnO_x = 20 mol%, 5 mL CH_3CN, 0.3 MPa O_2, 90 °C, 14 h.

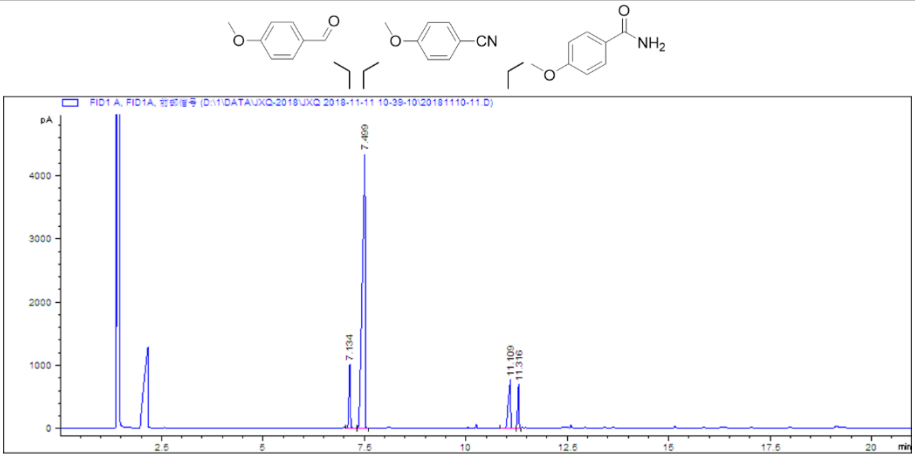

Supplementary Figure 9 GC spectrum of products from aerobic oxidation reaction of 4-methoxybenzylamine over MnO_x (**Figure 4** in the main text; **Supplementary Table 1, entry 1**). Reaction conditions: 1 mmol 4-methoxybenzylamine, 0.1 mmol MnO_x, 5 mL CH_3CN, 0.3 MPa O_2, 90 ^oC, 14 h.

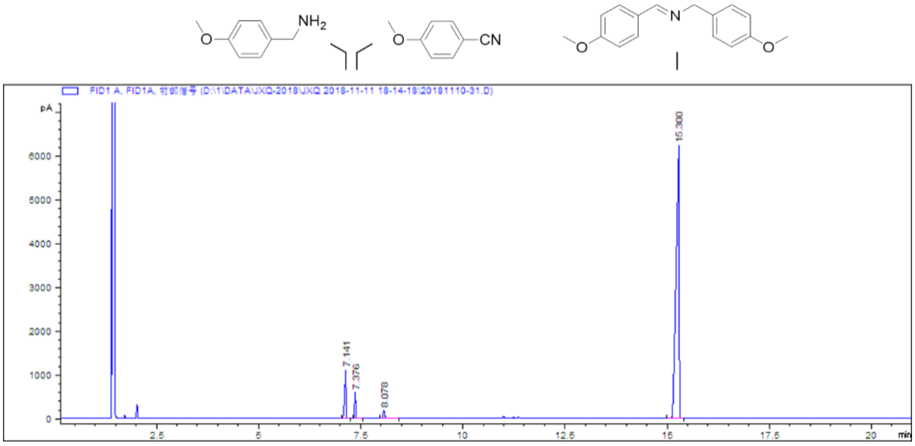

Supplementary Figure 10 GC spectrum of products from aerobic oxidation reaction of 4-methoxybenzylamine over acac-MnO_x (**Figure 4** in the main text; **Supplementary Table 1, entry 2**). Reaction conditions: 1 mmol 4-methoxybenzylamine, 0.1 mmol MnO_x, acac/MnO_x = 20 mol%, 5 mL CH_3CN, 0.3 MPa O_2, 90 ^oC, 14 h.

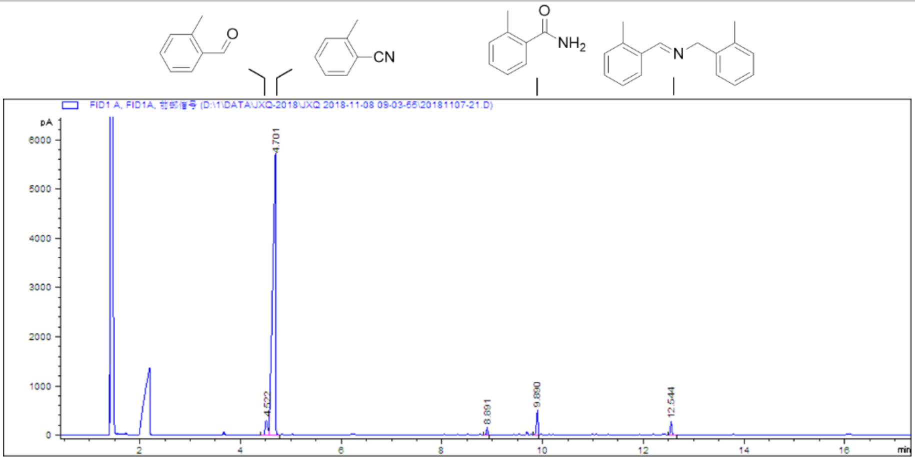

Supplementary Figure 11 GC spectrum of products from aerobic oxidation reaction of 2-methylbenzylamine over MnO_x (**Figure 4** in the main text; **Supplementary Table 1, entry 3**). Reaction conditions: 1 mmol 2-methylbenzylamine, 0.1 mmol MnO_x, 5 mL CH_3CN, 0.3 MPa O_2, 90 ^oC, 14 h.

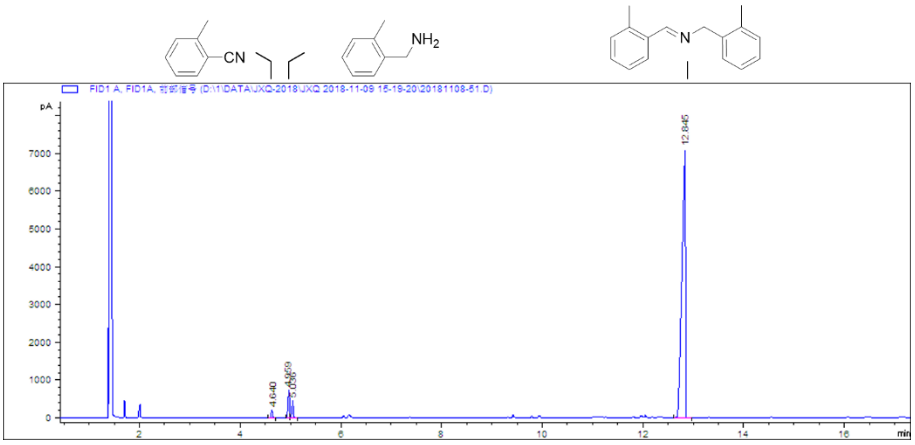

Supplementary Figure 12 GC spectrum of products from aerobic oxidation reaction of 2-methylbenzylamine over acac-MnO_x (**Figure 4** in the main text; **Supplementary Table 1, entry 4**). Reaction conditions: 1 mmol 2-methylbenzylamine, 0.1 mmol MnO_x, acac/MnO_x = 20 mol%, 5 mL CH_3CN, 0.3 MPa O_2, 90 ^oC, 14 h.

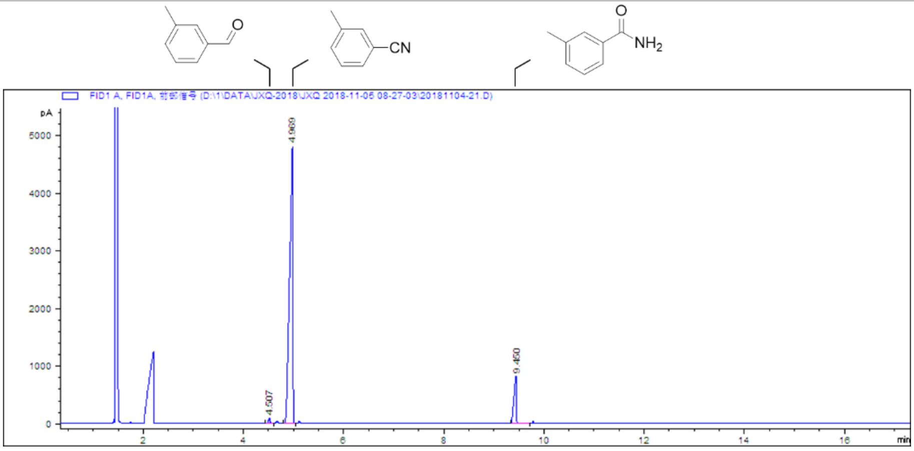

Supplementary Figure 13 GC spectrum of products from aerobic oxidation reaction of 3-methylbenzylamine over MnO_x (**Figure 4** in the main text; **Supplementary Table 1, entry 5**). Reaction conditions: 1 mmol 3-methylbenzylamine, 0.1 mmol MnO_x, 5 mL CH_3CN, 0.3 MPa O_2, 90 ^oC, 14 h.

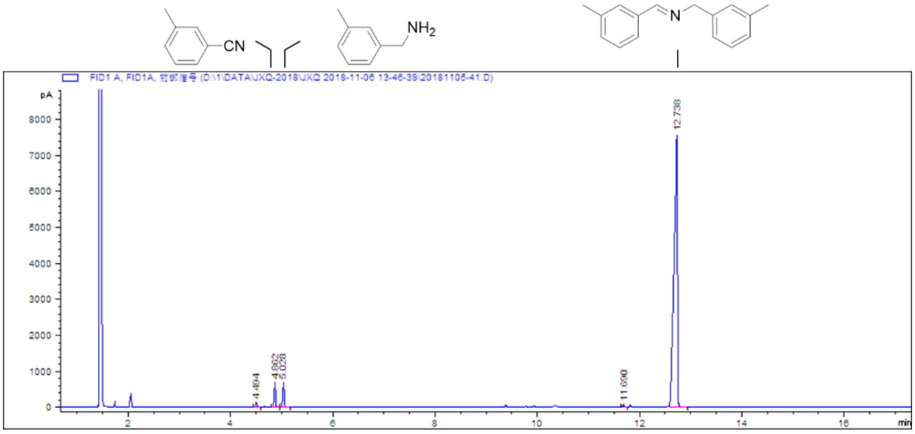

Supplementary Figure 14 GC spectrum of products from aerobic oxidation reaction of 3-methylbenzylamine over acac-MnO_x (**Figure 4** in the main text; **Supplementary Table 1, entry 6**). Reaction conditions: 1 mmol 3-methylbenzylamine, 0.1 mmol MnO_x, acac/MnO_x = 20 mol%, 5 mL CH_3CN, 0.3 MPa O_2, 90 ^oC, 14 h.

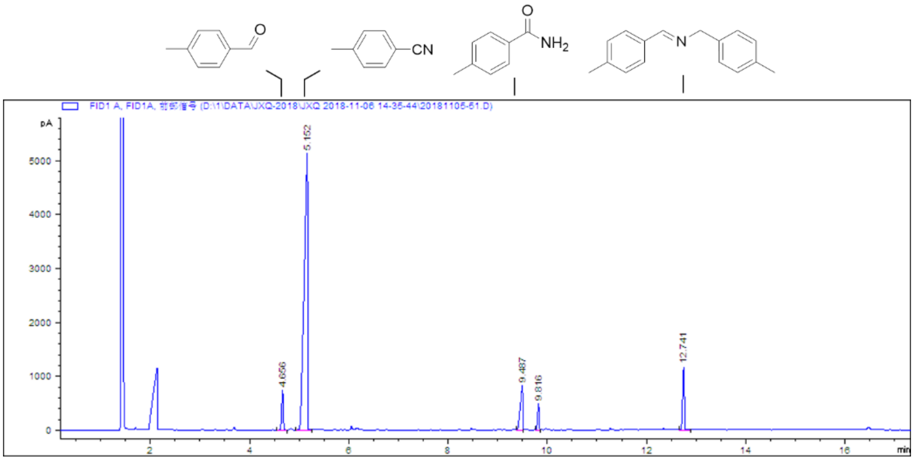

Supplementary Figure 15 GC spectrum of products from aerobic oxidation reaction of 4-methylbenzylamine over MnO_x (**Figure 4** in the main text; **Supplementary Table 1, entry 7**). Reaction conditions: 1 mmol 4-methylbenzylamine, 0.1 mmol MnO_x, 5 mL CH_3CN, 0.3 MPa O_2, 90 ^oC, 14 h.

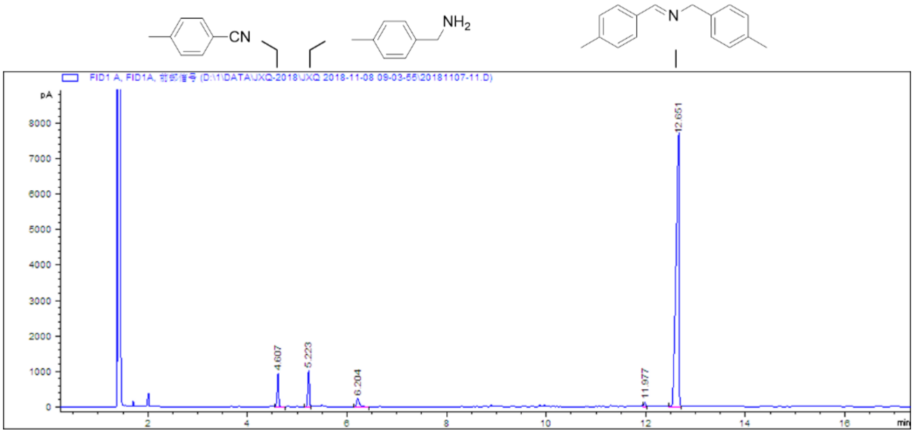

Supplementary Figure 16 GC spectrum of products from aerobic oxidation reaction of 4-methylbenzylamine over acac-MnO_x (**Figure 4** in the main text; **Supplementary Table 1, entry 8**). Reaction conditions: 1 mmol 4-methylbenzylamine, 0.1 mmol MnO_x, acac/MnO_x = 20 mol%, 5 mL CH_3CN, 0.3 MPa O_2, 90 ^oC, 14 h.

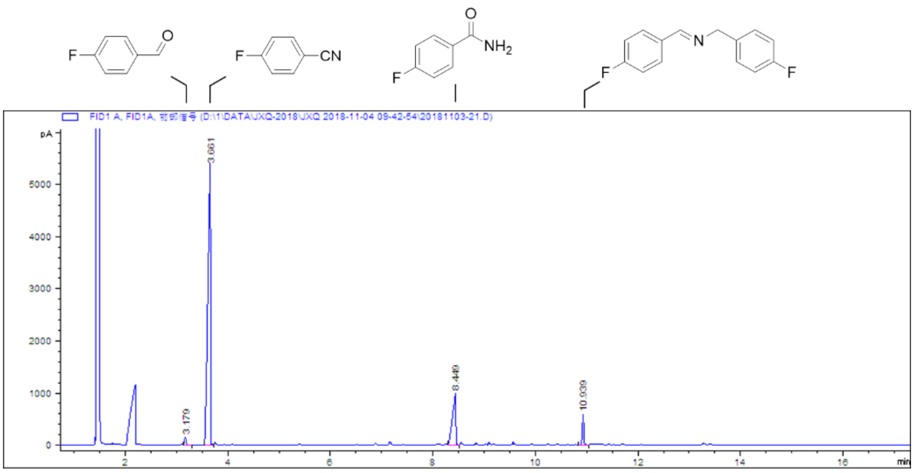

Supplementary Figure 17 GC spectrum of products from aerobic oxidation reaction of 4-fluorobenzylamine over MnO_x (**Figure 4** in the main text; **Supplementary Table 1, entry 9**). Reaction conditions: 1 mmol 4-fluorobenzylamine, 0.1 mmol MnO_x, 5 mL CH_3CN, 0.3 MPa O_2, 90 ^oC, 14 h.

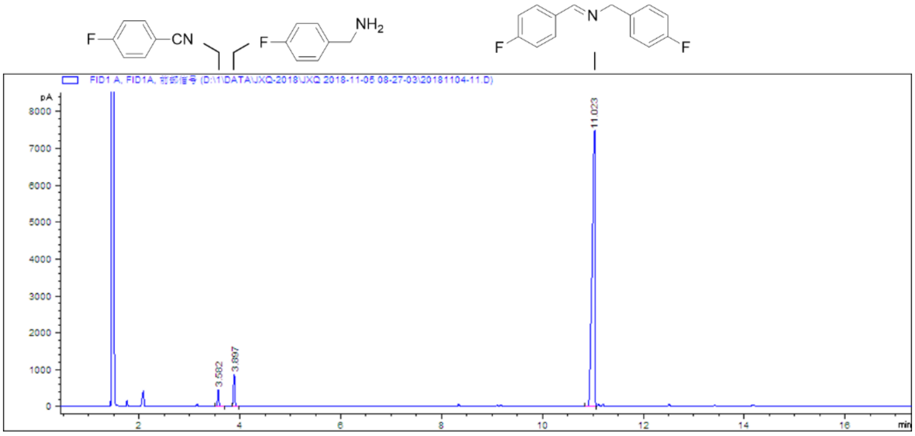

Supplementary Figure 18 GC spectrum of products from aerobic oxidation reaction of 4-fluorobenzylamine over acac-MnO_x (**Figure 4** in the main text; **Supplementary Table 1, entry 10**). Reaction conditions: 1 mmol 4-fluorobenzylamine, 0.1 mmol MnO_x, acac/MnO_x = 20 mol%, 5 mL CH_3CN, 0.3 MPa O_2, 90 ^oC, 14 h.

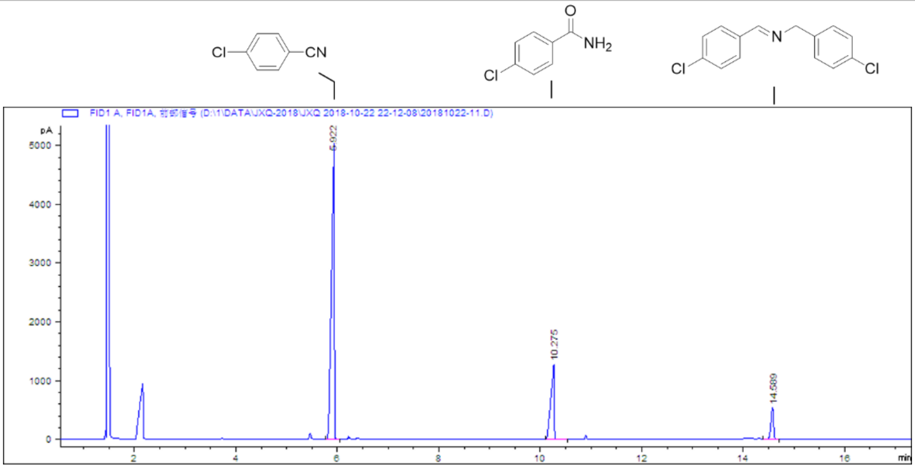

Supplementary Figure 19 GC spectrum of products from aerobic oxidation reaction of 4-chlorobenzylamine over MnO_x (**Figure 4** in the main text; **Supplementary Table 1, entry 11**). Reaction conditions: 1 mmol 4-chlorobenzylamine, 0.1 mmol MnO_x, 5 mL CH_3CN, 0.3 MPa O_2, 90 ^oC, 14 h.

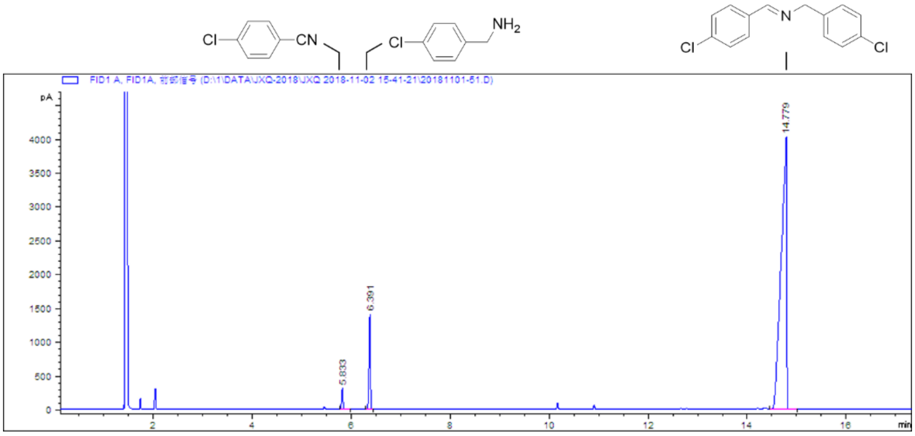

Supplementary Figure 20 GC spectrum of products from aerobic oxidation reaction of 4-chlorobenzylamine over acac-MnO_x (**Figure 4** in the main text; **Supplementary Table 1, entry 12**). Reaction conditions: 1 mmol 4-chlorobenzylamine, 0.1 mmol MnO_x, acac/MnO_x = 20 mol%, 5 mL CH_3CN, 0.3 MPa O_2, 90 ^oC, 14 h.

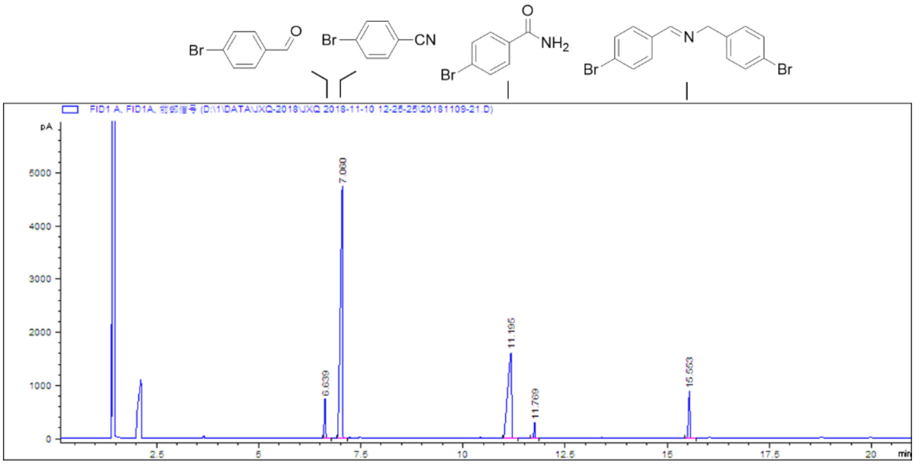

Supplementary Figure 21 GC spectrum of products from aerobic oxidation reaction of 4-bromobenzylamine over MnO_x (**Figure 4** in the main text; **Supplementary Table 1, entry 13**). Reaction conditions: 1 mmol 4-bromobenzylamine, 0.1 mmol MnO_x, 5 mL CH_3CN, 0.3 MPa O_2, 90 ^oC, 14 h.

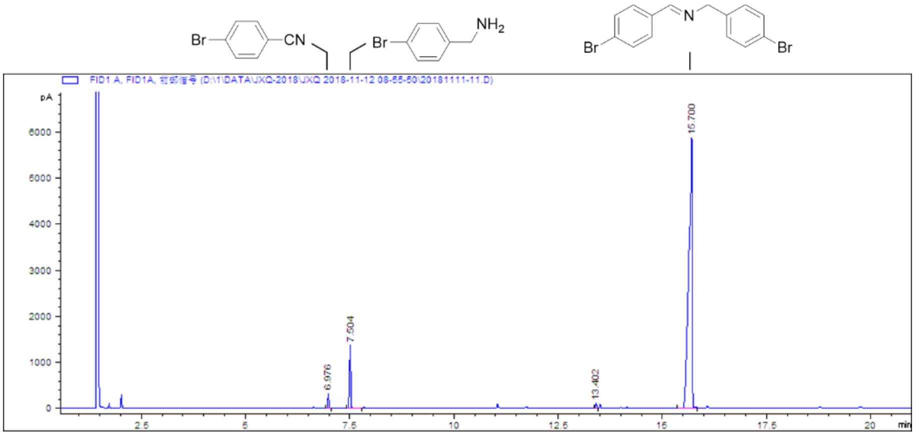

Supplementary Figure 22 GC spectrum of products from aerobic oxidation reaction of 4-bromobenzylamine over acac-MnO_x (**Figure 4** in the main text; **Supplementary Table 1, entry 14**). Reaction conditions: 1 mmol 4-bromobenzylamine, 0.1 mmol MnO_x, acac/MnO_x = 20 mol%, 5 mL CH_3CN, 0.3 MPa O_2, 90 ^oC, 14 h.

MS: m/z (%): 195 (33) [M+], 194 (35), 117 (11), 91 (100), 65 (15)

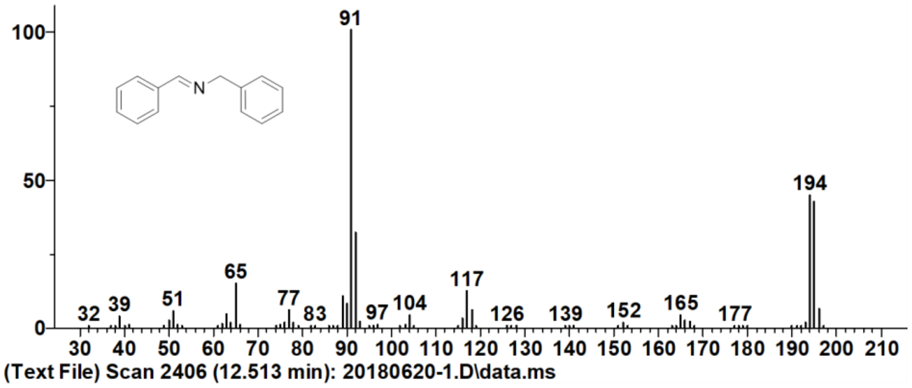

Supplementary Figure 23 Mass spectrum of **2a**.

MS: m/z (%): 103 (100) [M+], 76 (34), 50 (11), 32 (25)

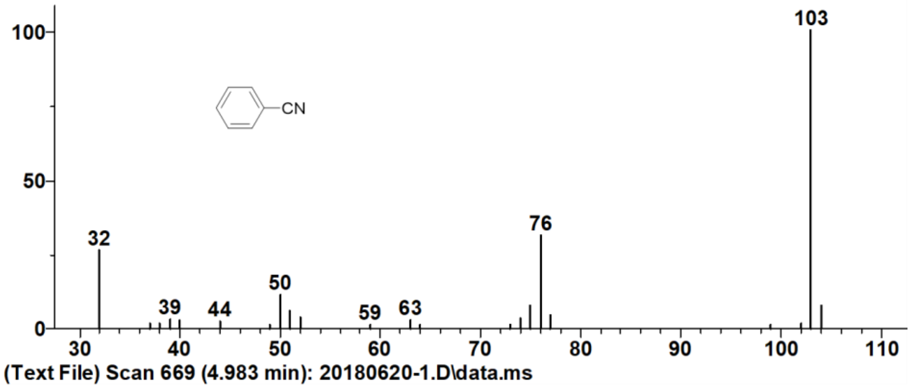

Supplementary Figure 24 Mass spectrum of **3a**.

MS: m/z (%): 255 (20) [M+], 147 (4), 121 (100), 91 (5), 77 (8)

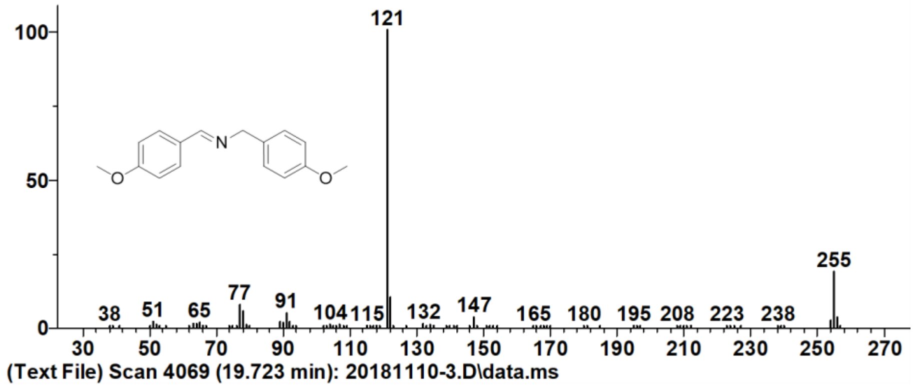

Supplementary Figure 25 Mass spectrum of **N-(4-methoxybenzylidene)-N-(4-methoxybenzyl)amine** (**2b**).

MS: m/z (%): 133 (100) [M+], 118 (10), 103 (35), 90 (41), 76 (8), 63 (12)

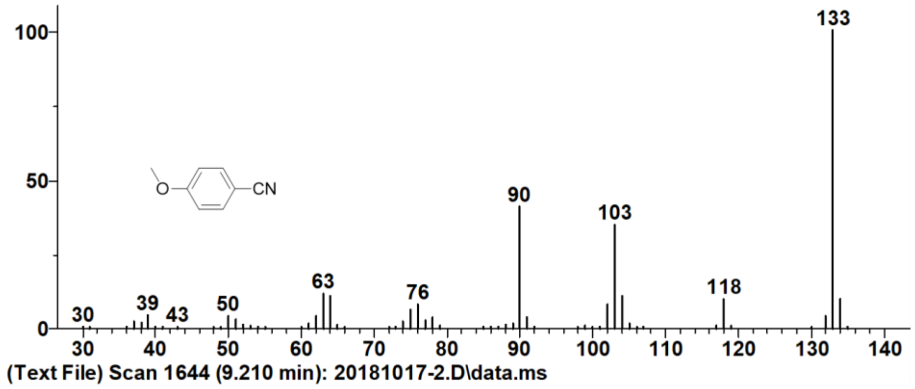

Supplementary Figure 26 Mass spectrum of **anisonitrile** (**3b**).

MS: m/z (%): 223 (44) [M+], 208 (8), 131 (10), 105 (100), 91 (8), 77 (13)

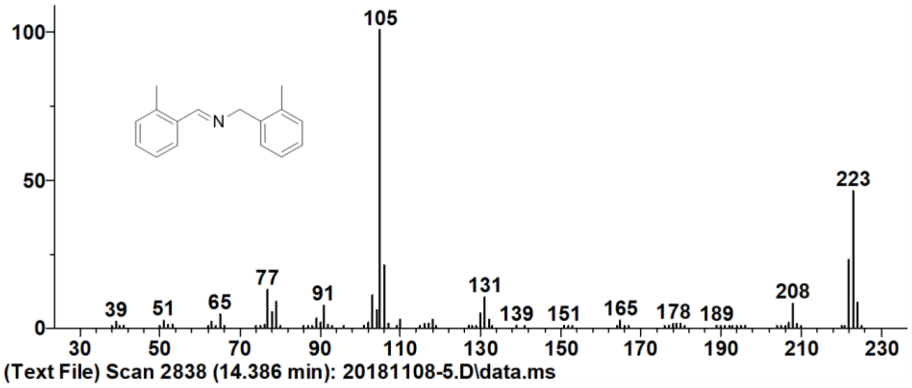

Supplementary Figure 27 Mass spectrum of **N-(2-methylbenzylidene)-N-(2-methylbenzyl)amine** (**2c**).

MS: m/z (%): 117 (100) [M+], 90 (41), 63 (12), 39 (6)

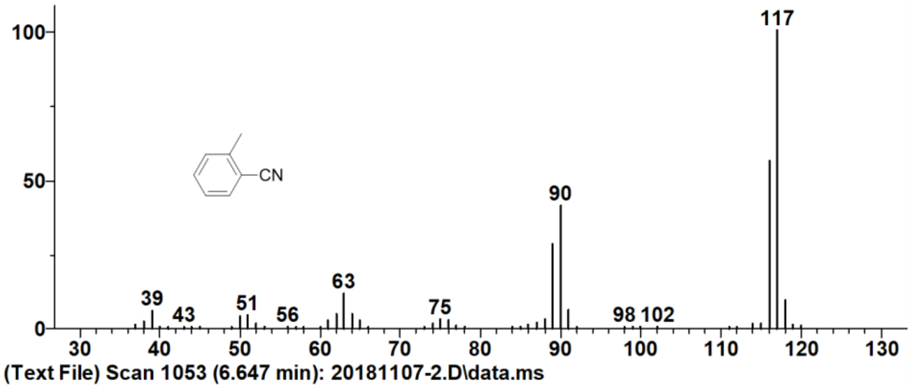

Supplementary Figure 28 Mass spectrum of **2-methylbenzonitrile**(**3c**).

MS: m/z (%): 223 (56) [M+], 208 (23), 131 (10), 105 (100), 91 (16), 77 (19)

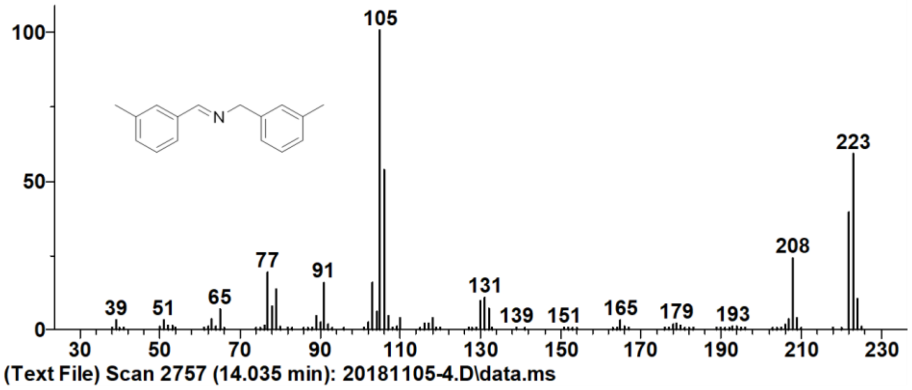

Supplementary Figure 29 Mass spectrum of **N-(3-methylbenzylidene)-N-(3-methylbenzyl)amine**(**2d**).

MS: m/z (%): 117 (100) [M+], 90 (37), 63 (10), 39 (6)

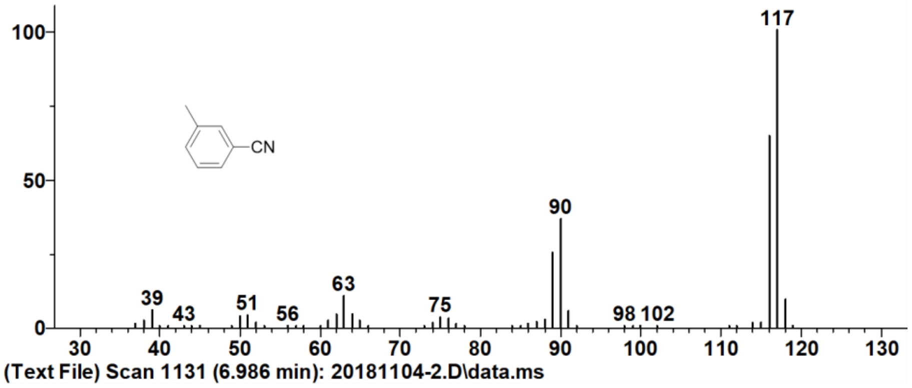

Supplementary Figure 30 Mass spectrum of **3-methylbenzonitrile**(**3d**).

MS: m/z (%): 223 (60) [M+], 208 (19), 118 (25), 105 (100), 91 (12), 77 (24)

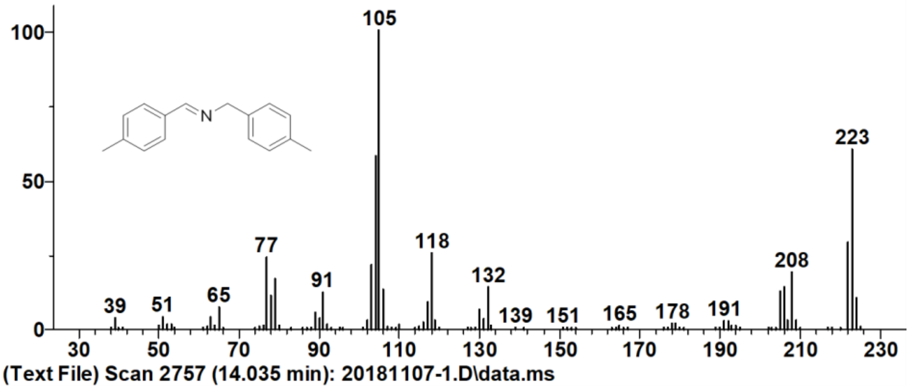

Supplementary Figure 31 Mass spectrum of **N-(4-methylbenzylidene)-N-(4-methylbenzyl)amine**(**2e**).

MS: m/z (%): 117 (100) [M+], 90 (34), 63 (10), 50 (4)

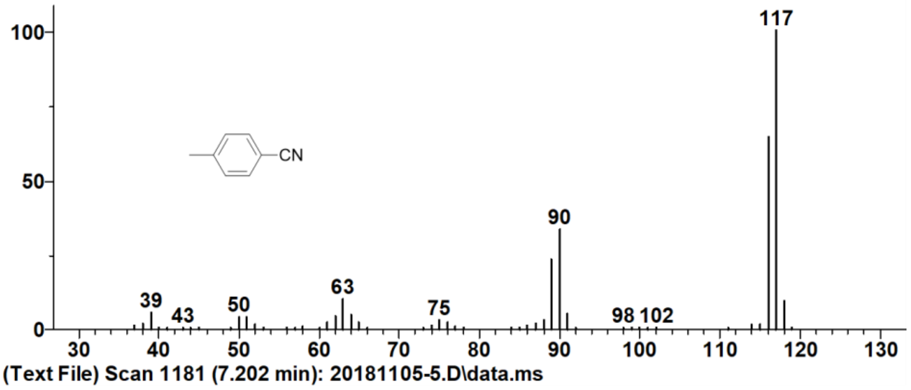

Supplementary Figure 32 Mass spectrum of **4-methylbenzonitrile**(**3e**).

MS: m/z (%): 231 (31) [M+], 135 (8), 109 (100), 83 (12)

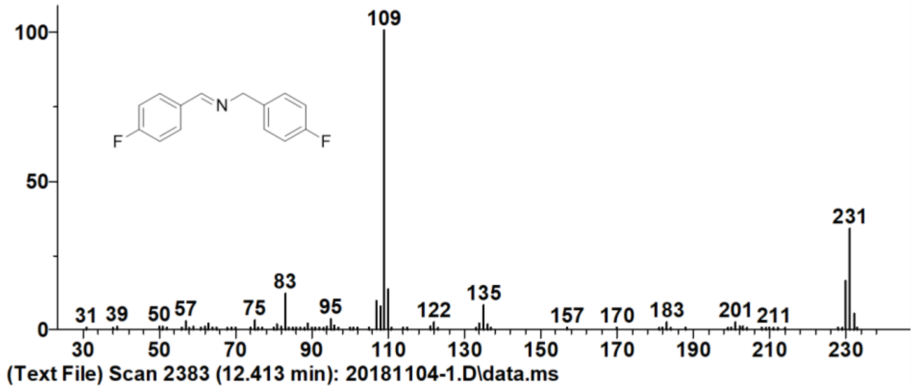

Supplementary Figure 33 Mass spectrum of **N-(4-fluorobenzylidene)-N-(4-fluorobenzyl)amine**(**2f**).

MS: m/z (%): 121 (100) [M+], 94 (31), 75 (6), 50 (5)

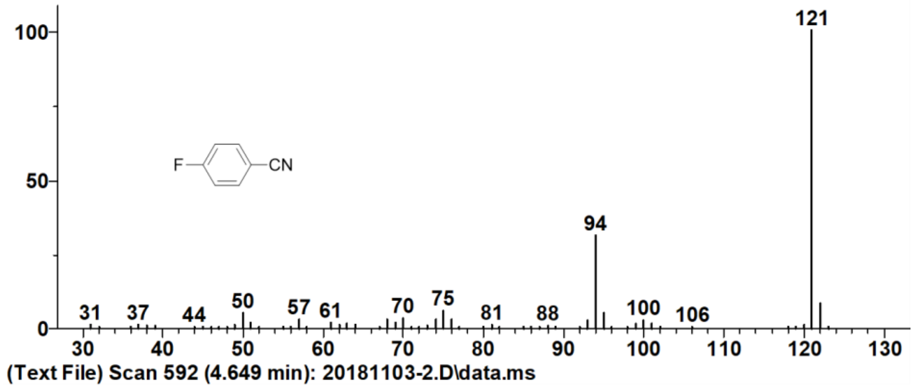

Supplementary Figure 34 Mass spectrum of **4-fluorobenzonitrile**(**3f**).

MS: m/z (%): 265 (17) [M+], 263 (26) [M+], 151 (11), 125 (100), 89 (22)

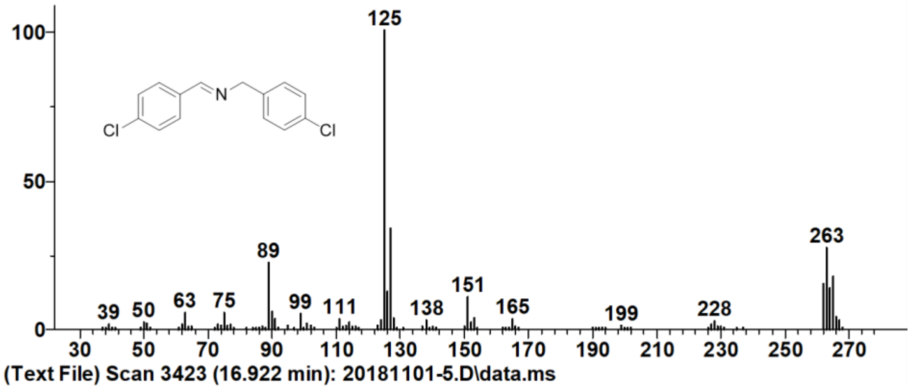

Supplementary Figure 35 Mass spectrum of **N-(4-chlorobenzylidene)-N-(4-chlorobenzyl)amine**(**2g**).

MS: m/z (%): 139 (35) [M+], 137 (100) [M+], 102 (30), 75 (13), 50 (8)

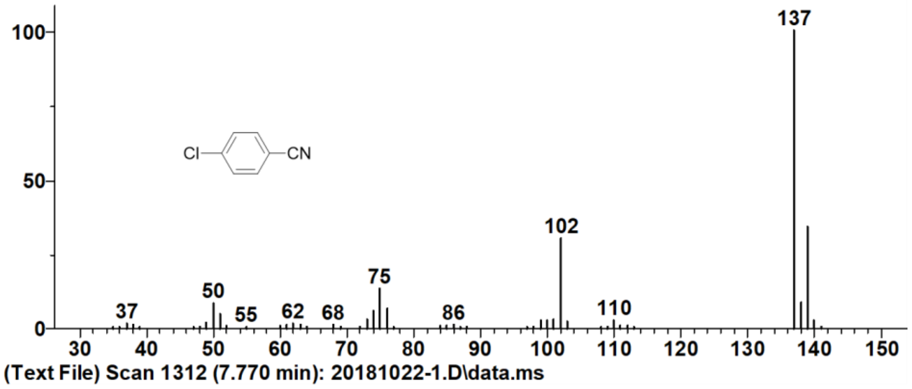

Supplementary Figure 36 Mass spectrum of **4-chlorobenzonitrile**(**3g**).

MS: m/z (%): 353 (39) [M+], 272 (5), 195 (8), 169 (100), 89 (36), 63 (9)

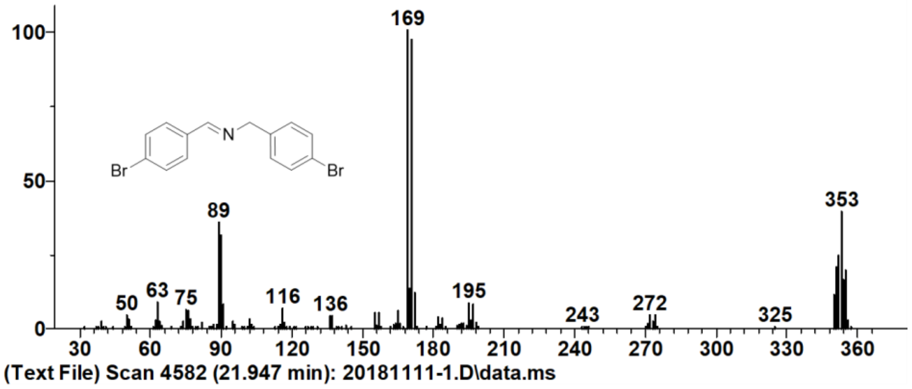

Supplementary Figure 37 Mass spectrum of **N-(4-bromobenzylidene)-N-(4-bromobenzyl)amine**(**2h**).

MS: m/z (%): 183 (96) [M+], 181 (100) [M+], 102 (89), 75 (26), 50 (15)

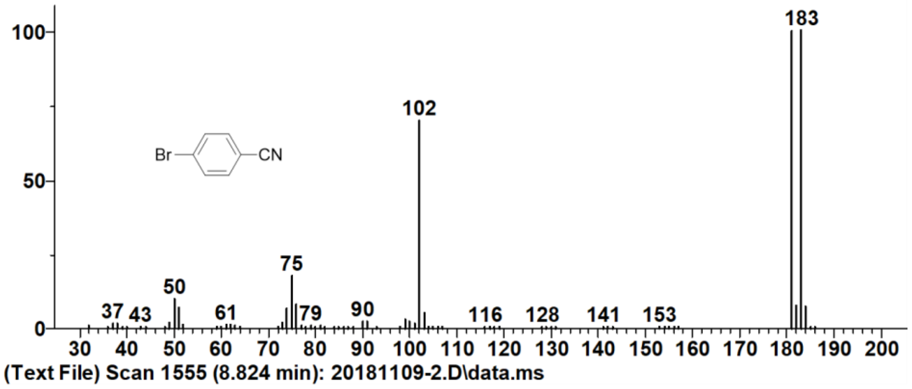

Supplementary Figure 38 Mass spectrum of **4-bromobenzonitrile**(**3h**).

Supplementary References

[1] Jia, X., Ma, J., Wang, M., Li, X., Gao, J. & Xu, J. Alkali α-MnO_2/Na_xMnO_2 collaboratively catalyzed ammoxidation-Pinner tandem reaction of aldehydes. *Catal. Sci. Technol.* **6**, 7429-7436 (2016).

[2] Ghodbane, O., Pascal, J.-L. & Favier, F. Microstructural effects on charge-storage properties in MnO_2-based electrochemical supercapacitors. *ACS Appl. Mater. Interfaces* **1**, 1130-1139 (2009).

[3] DeGuzman, R. N., *et al.* Synthesis and characterization of octahedral molecular sieves (OMS-2) having the hollandite structure. *Chem. Mater.* **6**, 815-821 (1994).
