# Supplementary material for: Switching acidity on manganese oxide catalyst with acetylacetones for selectivity-tunable amines oxidation
Source: Nat Commun. 2019 May 28;10:2338. doi: 10.1038/s41467-019-10315-9 (PMC6538668; doi:10.1038/s41467-019-10315-9)
Supplement: Supplementary file 2 — Peer Review [file 41467_2019_10315_MOESM2_ESM.pdf]

Reviewers' comments:

Reviewer #1 (Remarks to the Author):

This manuscript reports on a study of the use of organic modifiers on MnOx to control selectivity during the oxidation of amines to imines or nitriles. The authors observe that use of modifiers like "acac", in which two carbonyl functions can coordinate to the surface, can lead to much enhanced selectivity toward imines. The authors used reactivity studies together with surface vibrational spectroscopy to attribute the improved selectivity to the selective poisoning of particular Lewis acid sites on the surface. The authors showed that the main feature of the ligands that was important was having the di-carbonyl structure. Overall, this is an interesting example of using organic modifiers to control surface chemistry, and I think the manuscript is publishable in Nature Comm. I have a few comments for the authors to consider:

(1) The authors do not seem to consistently report experimental uncertainties in their results, for example in the measurement of reaction rates. Preferably these uncertainties would be obtained from separately-prepared batches of the modified catalysts.
(2) Can the authors provide some estimate of the surface coverage of the organic modifiers?
(3) The case for the selective poisoning could be strengthened by carrying out FTIR studies after pyridine adsorption for some of the modifiers that showed intermediate selectivity in Figure 1. Does one see some retention of Lewis acids in those materials?

Reviewer #2 (Remarks to the Author):

This is a nice paper that studies the use of acac modification of MnOx catalysts for controlled benzylamine arobic oxidation. It is shown that with acac species that form enolic forms on the MnOx surface that the Lewis acid sites are blocked and the oxidation of benzylamine can be stopped at imine, rather than being converted to the nitrile. The paper is overall well done and publication is recommended after some issues are addressed.

1. The surface area of amorphous MnOx vs. crystalline MnO2 should be given. Otherwise the statement that the amorphous catalyst is more active is meaningless.
2. On page 2 it is stated: "These results suggest that the tuning of the selectivity dose not result from the steric effect of the acac derivatives." This should be edited as it is ultimately shown that the acac species are sterically blocking the Lewis acid site. So this sentence can be confusing.
3. The coverage of species in figure 1 on the surface should be measured and quoted? TGA should be done. Is the coverage similar for all species? What is the limit of stability?
4. Keto vs enol form is discussed in context of directing selectivity. Is this just based on the stability of the produced surface bound species under reaction conditions? Ie. are the enolic species generally bound more strongly than keto species? This should be examined and discussed.

Response to referees

Manuscript ID.: NCOMMS-18-38429A

Title: Switching Acidity on Manganese Oxide Catalyst with Acetylacetones for Selectivity-tunable Amines Oxidation

Referee: 1

This manuscript reports on a study of the use of organic modifiers on MnO_x to control selectivity during the oxidation of amines to imines or nitriles. The authors observe that use of modifiers like "acac", in which two carbonyl functions can coordinate to the surface, can lead to much enhanced selectivity toward imines. The authors used reactivity studies together with surface vibrational spectroscopy to attribute the improved selectivity to the selective poisoning of particular Lewis acid sites on the surface. The authors showed that the main feature of the ligands that was important was having the di-carbonyl structure. Overall, this is an interesting example of using organic modifiers to control surface chemistry, and I think the manuscript is publishable in Nature Comm. I have a few comments for the authors to consider:

Comment 1: The authors do not seem to consistently report experimental uncertainties in their results, for example in the measurement of reaction rates. Preferably these uncertainties would be obtained from separately-prepared batches of the modified catalysts.

Response: Thank you for the reviewer's comments and constructive suggestions. As shown in Table R1 and the revised manuscript, the estimates of uncertainties have been added to the data in Table 1 after repeat measurements.

Table R1 The catalytic performance of manganese oxide catalysts in the benzylamine aerobic oxidation reaction.

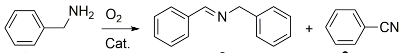

1a 2a 3a

Entry	Cat.	Mass-specific activity (mmol g_{cat}^{-1} h^{-1})	Conv. (%)	Yield (%)	
				**2a**	**3a**
**1**	α-MnO_2	45.4±2.1	94.5±0.4	47.9±2.4	44.6±3.1
**2**	γ-MnO_2	61.8±4.0	97.4±0.2	58.6±4.4	32.5±3.7
**3**	δ-MnO_2	15.7±0.7	94.9±0.1	61.9±3.0	24.6±5.0
**4**	OMS-2	63.4±5.2	93.1±0.2	24.1±2.6	58.9±2.0
**5**	MnO_x	129.2±4.2	>99	0	86.5±0.3
**6**[a]	MnO_x	-	>99	7.0±1.2	80.1±1.0
**7**[a, b]	MnO_x	-	93.0±0.2	46.7±0.2	41.2±2.5
**8**	acac-MnO_x	14.7±1.9	94.1±0.3	90.6±4.1	3.4±0.2

Reaction conditions: 1 mmol Benzylamine, 0.1 mmol catalyst, acac/MnO_x = 20 mol% for acac-MnO_x, 5 mL CH_3CN, 0.3 MPa O_2, 90 °C, 14 h. [a] 0.05 mmol MnO_x. [b] 60 °C.

Comment 2: Can the authors provide some estimate of the surface coverage of the organic modifiers?

Response: Owing to the convenience of analyzing the organic modifiers in solution by GC, we measured the loss of modifiers from the solutions after adsorption on MnO_x. And the surface coverages were calculated by dividing the depletion of modifiers from the solution by the surface area of the MnO_x catalyst (Fig. R1). Surface coverages at 5-6 μmol m^{-2} were observed for the modifiers of acac (**1**), 2-acetylcyclohexanone (**2**), 3-butyl-2,4-pentanedione (**3**), 3-methyl-2,4-pentanedione (**4**) and 8-hydroxyquinoline (**5**). By contrast, a considerably low surface coverage was observed over pyridine (**6**) or 3,3-dimethyl-2,4-pentanedione (**7**) modified MnO_x. This is in accordance with the performance of MnO_x modified by these modifiers in the aerobic oxidation of benzylamine (Fig. R3). The adsorptive property of acac (**1**) and 3,3-dimethyl-2,4-pentanedione (**7**) on MnO_x was further investigated at various concentrations (Fig. R2). The result showed that the adsorption of acac on MnO_x was nearly complete in the range of 0-20 mol% of acac and increased slowly in range above 20 mol% which indicated the excess of unadsorbed acac modifier in the solution. While the surface coverage of 3,3-dimethyl-2,4-pentanedione (**7**) on MnO_x seemed to be constantly low at tested concentrations.

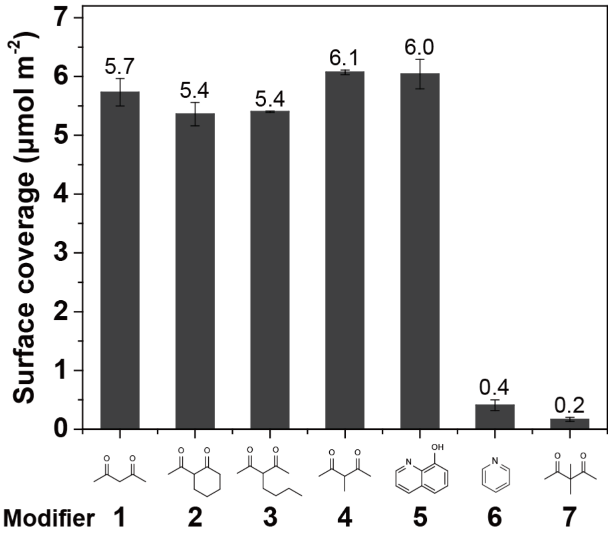

Fig. R1 Surface coverage of the organic modifiers. Process for evaluating the modifier adsorption on MnO_x: Immersing 0.1 mmol MnO_x in a 5 mL acetonitrile solution of modifiers (modifier/MnO_x = 20 mol%). After stirring at 90 °C for 4 h, the excess of unadsorbed modifier in solution was determined by GC analysis.

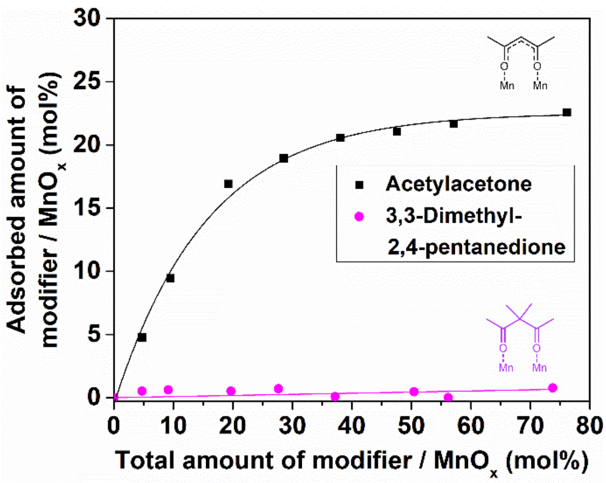

Fig. R2 The chart of the acetylacetone and 3,3-dimethyl-2,4-pentanedione adsorption on MnO_x. Process for evaluating the modifier adsorption on MnO_x: Immersing 0.1 mmol MnO_x in a 5 mL acetonitrile solution of modifiers. After stirring at 90 °C for 4 h, the excess of unadsorbed modifier in solution was determined by GC analysis.

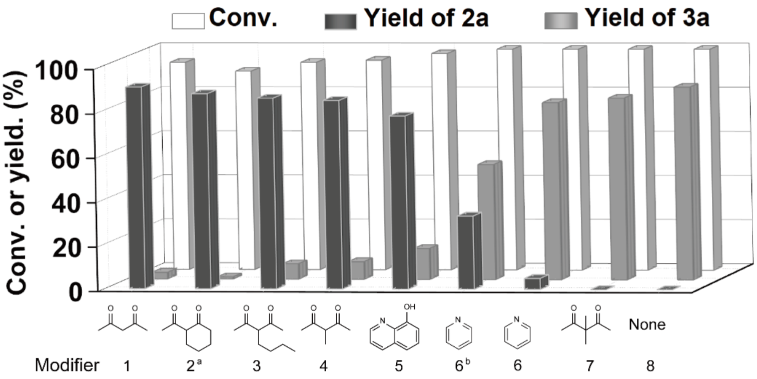

Fig. R3 (**Fig. 1** in the manuscript) Performance of MnO_x with different modifiers in the aerobic oxidation of benzylamine. Reaction conditions: 1 mmol benzylamine, 0.1 mmol MnO_x, additive/MnO_x = 20 mol%, 5 mL CH_3CN, 0.3 MPa O_2, 90 °C, 14 h. [a] 20 h. [b] Additive/MnO_x = 200 mol%.

Comment 3: The case for the selective poisoning could be strengthened by carrying out FTIR studies after pyridine adsorption for some of the modifiers that showed intermediate selectivity in Figure 1. Does one see some retention of Lewis acids in those materials?

Response: It is reasonable to expect the retention of Lewis acidity in those modified catalysts which showed low selectivity for imine. As shown in Fig. R4 and Table R2, Fourier transform infrared (FT-IR) spectroscopic characterization of pyridine

adsorption confirmed the preservation of Lewis acidity on 3,3-dimethyl-2,4-pentanedione (**7**) modified MnO_x, which gave nitrile as the primary product in the aerobic oxidation of benzylamine. To examine the acid property of catalysts showing intermediate selectivity, a decreased amount of 10 mol% acac modified MnO_x was prepared and tested. As shown in Fig. R4 and Table R2, a residual Lewis acidity was observed for 10 mol% acac modified MnO_x, and a medium yield (51.0%) of imine (**2a**) was obtained over 10 mol% acac modified MnO_x under identical conditions. By contrast, after pre-adsorption of 20 mol% acac on MnO_x, the band at around 1440 cm^{-1} was scarcely observed, and the product of imine could be selectively obtained over 20 mol% acac modified MnO_x.

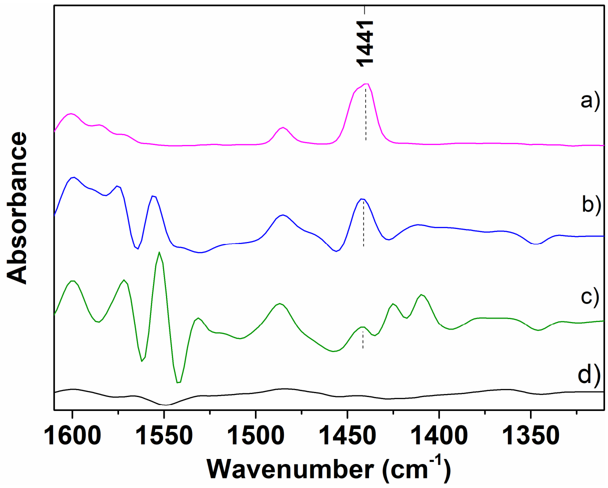

Fig. R4 FT-IR spectra of pyridine adsorption to a) clean MnO_x, b) 20 mol% 3,3-Dimethyl-2,4-pentanedione modified MnO_x, c) 10 mol% acac modified MnO_x and d) 20 mol% acac modified MnO_x.

Table R2 The catalytic performance of different manganese oxide catalysts in the benzylamine aerobic oxidation reaction.

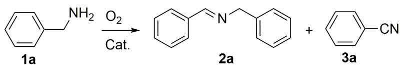

Entry	Cat.	Conv. (%)	Yield (%)	
			2a	3a
**1**	a in Fig. R4	>99	0	86.5
**2**	b in Fig. R4	>99	0	82.1
**3**	c in Fig. R4	96.3	51.0	42.5
**4**	d in Fig. R4	94.1	90.6	3.4

Reaction conditions: 1 mmol benzylamine, 0.1 mmol MnO_x, 5 mL CH_3CN, 0.3 MPa O_2, 90 °C, 14 h.

Referee: 2

This is a nice paper that studies the use of acac modification of MnO_x catalysts for controlled benzylamine aerobic oxidation. It is shown that with acac species that form enolic forms on the MnO_x surface that the Lewis acid sites are blocked and the oxidation of benzylamine can be stopped at imine, rather than being converted to the nitrile. The paper is overall well done and publication is recommended after some issues are addressed.

Comment 1: The surface area of amorphous MnO_x vs. crystalline MnO_2 should be given. Otherwise the statement that the amorphous catalyst is more active is meaningless.

Response: Thank you for the reviewer's comments and constructive suggestions. It is a challenge to determine the intrinsic reactivity of metal oxide catalysts due to the lack of enough insight into the active sites. We found that amorphous MnO_x showed the highest mass-specific activity, which is a general indicator for the performance of catalyst. We further measured the specific surface area of amorphous MnO_x vs. crystalline MnO_2 according to the reviewer's comments. As shown in Table R3, the surface-specific activity of amorphous MnO_x is much lower than that of α-MnO_2. Therefore, in the revised manuscript, we took out the statement that the amorphous catalyst is the most active.

Table R3 The result of surface area and activity for amorphous MnO_x vs. crystalline MnO_2.

Entry	Cat.	BET surface area (m^2 g^{-1})	Mass-specific activity (mmol g_{cat}^{-1} h^{-1})	Surface-specific activity (mmol h^{-1} m^{-2})
1	α-MnO_2	28.2	45.4±2.1	1.6±0.1
2	γ-MnO_2	69.5	61.8±4.0	0.9±0.1
3	δ-MnO_2	49.7	15.7±0.7	0.3
4	OMS-2	77.4	63.4±5.2	0.8±0.1
5	MnO_x	250.4	129.2±4.2	0.5

Comment 2: On page 2 it is stated: "These results suggest that the tuning of the selectivity dose not result from the steric effect of the acac derivatives." This should be edited as it is ultimately shown that the acac species are sterically blocking the Lewis acid site. So this sentence can be confusing.

Response: We are sorry for this confusing statement. As shown in the revised manuscript, we edited this sentence as "These results suggest that the acac derivatives with different substituent groups are effective modifiers for tuning the selectivity".

Comment 3: The coverage of species in Figure 1 on the surface should be measured and quoted? TGA should be done. Is the coverage similar for all species? What is the limit of stability?

Response: Owing to the convenience of analyzing the organic modifiers in solution by GC, we measured the loss of modifiers from the solutions after adsorption on MnO_x. And the surface coverages were calculated by dividing the depletion of modifiers from the solution by the surface area of the MnO_x catalyst (Fig. R1). Similar surface coverages were observed for the modifiers of acac (**1**), 2-acetylcyclohexanone (**2**), 3-butyl-2,4-pentanedione (**3**), 3-methyl-2,4-pentanedione (**4**) and 8-hydroxyquinoline (**5**). By contrast, a considerably low surface coverage was observed over pyridine (**6**) or 3,3-dimethyl-2,4-pentanedione (**7**) modified MnO_x. The adsorptive property of acac (**1**) and 3,3-dimethyl-2,4-pentanedione (**7**) on MnO_x was further investigated at various concentrations (Fig. R2). The result showed that the adsorption of acac on MnO_x was nearly complete in the range of 0-20 mol% of acac and increased slowly in range above 20 mol% which indicated the excess of unadsorbed acac modifier in the solution. While the surface coverage of 3,3-dimethyl-2,4-pentanedione (**7**) on MnO_x seemed to be constantly low at tested concentrations.

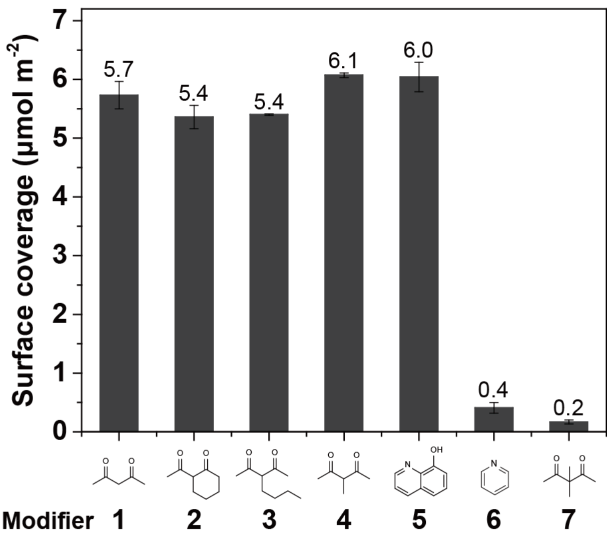

Fig. R1 Surface coverage of the organic modifiers. Process for evaluating the modifier adsorption on MnO_x: Immersing 0.1 mmol MnO_x in a 5 mL acetonitrile solution of modifiers (modifier/MnO_x = 20 mol%). After stirring at 90 ºC for 4 h, the excess of unadsorbed modifier in solution was determined by GC analysis.

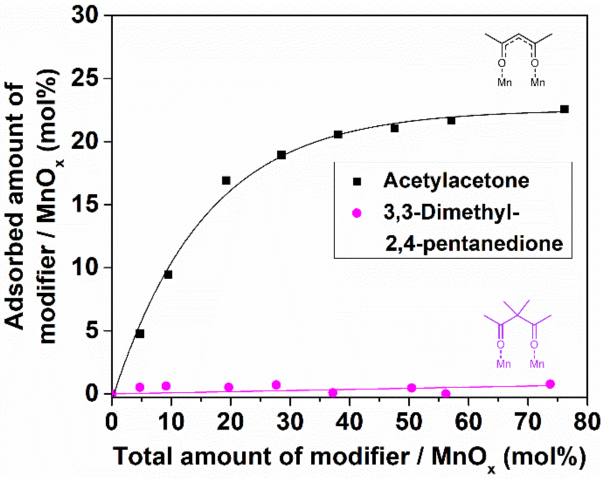

Fig. R2 The chart of the acetylacetone and 3,3-dimethyl-2,4-pentanedione adsorption on MnO_x. Process for evaluating the modifier adsorption on MnO_x: Immersing 0.1 mmol MnO_x in a 5 mL acetonitrile solution of modifiers. After stirring at 90 ºC for 4 h, the excess of unadsorbed modifier in solution was determined by GC analysis.

The results of TGA analysis of clean and 20 mol% acac modified MnO_x materials are shown in Fig. R5. The first weight loss is before 200 °C, owing to desorption of physisorbed H_2O molecules for clean MnO_x, and desorption of physisorbed species of H_2O and acetonitrile solvent for 20 mol% acac modified MnO_x. The release of chemically adsorbed water may cause the slow weight loss between 200 and 500 °C for clean MnO_x (*ACS Sustainable Chem. Eng.* **3**, 2933-2943 (2015)). Likewise, the

relatively rapid weight loss between 200 and 500 °C for 20 mol% acac modified MnO_x should result from the release of chemically adsorbed water and acac. Thus, according to the TGA profiles, the mass fraction of chemically adsorbed acac was obtained. And the surface coverage of chemically adsorbed acac could be further calculated to be 6.0 μmol m^{-2}, which is approximate to the result calculated via GC analysis (Fig. R1). This provides a check of the utility of the GC measurement for determination of surface coverage of organic modifiers.

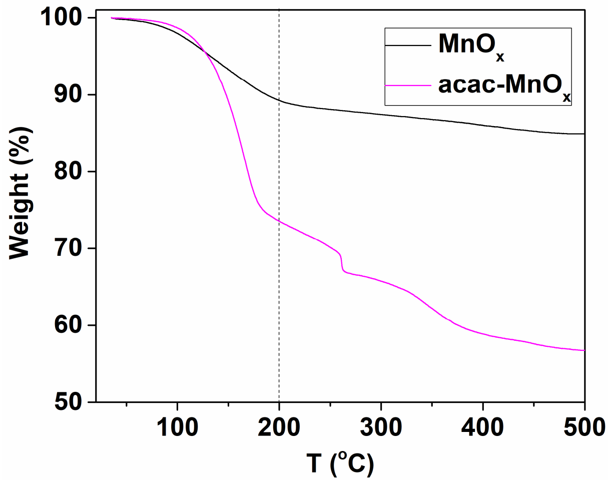

Fig. R5 TGA profiles of MnO_x and acac-MnO_x, respectively.

Based on the above results, it can be concluded that acac could modify the surface of MnO_x in considerable stability. In comparison, the sites for stable coordination with pyridine(**6**) or 3,3-dimethyl-2,4-pentanedione (**7**) are scarce on MnO_x. Acac and its derivatives are frequently found to be deprotonated to the corresponding anion and form stable adsorption on metal oxide surfaces (*J. Am. Chem. Soc.* **130**, 14329-14338 (2008); *J. Am. Chem. Soc.* **133**, 9014-9022 (2011); *Langmuir* **27**, 3442-3450 (2011)). The resonance forms for the enolate anion provides the major coordination mode (*J. Chem. Educ.*, **66**, 779-780 (1989)). In this form, the anion acts as a bidentate chelate, forming a stable ring on the metal oxide surface via charge delocalization (Fig. R2). By contrast, pyridine (**6**) is monodentate ligand, and 3,3-dimethyl-2,4-pentanedione (**7**) could not form stable bidentate chelate due to the lack of charge delocalization (Fig. R2; *J. Chem. Educ.*, **66**, 779-780 (1989)), which might account for the scarce modification of these two modifiers.

Comment 4: Keto vs enol form is discussed in context of directing selectivity. Is this just based on the stability of the produced surface bound species under reaction conditions? Ie. are the enolic species generally bound more strongly than keto species? This should be examined and discussed.

Response: The enol form is generally considered to be less stable in comparison with the keto form. However, in the case of acac, the enol tautomer is the most stable one (ca. 94% population under normal gas phase conditions) owing to the stabilizing effect of an internal hydrogen bond coupled with an electronic delocalization over the O-C-C-C-O pseudo cycle (*Phys. Chem. Chem. Phys.* **14**, 3450-3459 (2012)). Herein, we have also probed the free acac and acac modified on MnO_x via IR spectroscopy (Fig. R6). For free acac, two C=O stretching bands at 1728 and 1709 cm^{-1}, one C=C stretching band at 1625 cm^{-1} and two CH_3 bending vibration bands at 1421 and 1360 cm^{-1} were observed, all of which are attributed to enolic acac with varied conformations (*J. Phys. Chem. A* **111**, 3074-3081 (2007)). After the acac deposition on MnO_x, the C=O stretching bands attributed to free acac disapeared, and two bands were observed at 1556 and 1342 cm^{-1} that arise from the metal-complexed CO/CC stretching and CH_3 symmetrical bending vibration of coordinated acac, respectively (*J. Chem. Phys.* **46**, 1506-1510 (1967); *J. Organomet. Chem.* **772-773**, 222-228 (2014)). This indicates the formation of bidentate ligand from the enol anion form of acac via deprotonation and subsequent coordination on the surface of MnO_x. This is in accordance with previously reported enolate anion modes of acac coordinating on metal oxides (*J. Am. Chem. Soc.* **130**, 14329-14338 (2008); *J. Am. Chem. Soc.* **133**, 9014-9022 (2011); *Langmuir* **27**, 3442-3450 (2011)).

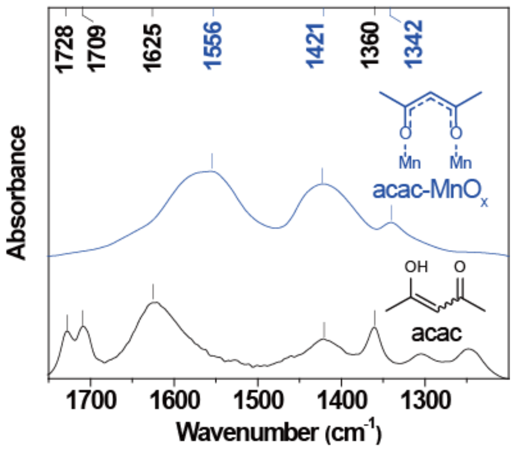

Fig. R6 (**Fig. 2a** in the manuscript) FT-IR spectra of acac and acac-MnO_x after evacuation at 150 ºC for 0.5 h.

REVIEWERS' COMMENTS:

Reviewer #1 (Remarks to the Author):

The authors have done a nice job of responding to the referee comments. I recommend publication.

Reviewer #2 (Remarks to the Author):

The authors reasonably addressed the referee concerns. The paper is now recommended for publication.
